# Hypoxia induces an early primitive streak signature, enhancing spontaneous elongation and lineage representation in gastruloids

**DOI:** 10.1242/dev.200679

**Published:** 2022-09-14

**Authors:** Natalia López-Anguita, Seher Ipek Gassaloglu, Maximilian Stötzel, Adriano Bolondi, Deniz Conkar, Marina Typou, René Buschow, Jesse V. Veenvliet, Aydan Bulut-Karslioglu

**Affiliations:** ^1^ Department of Genome Regulation, Max Planck Institute for Molecular Genetics, 14195 Berlin, Germany; ^2^ Institute of Chemistry and Biochemistry, Department of Biology, Chemistry and Pharmacy, Freie Universität Berlin, 14195 Berlin, Germany; ^3^ Stembryogenesis Lab, Max Planck Institute of Molecular Cell Biology and Genetics, 01307 Dresden, Germany; ^4^ Medical School, Democritus University of Thrace, 681 00 Alexandroupoli, Greece; ^5^ Department of Biomedical Sciences, International Hellenic University, 570 01, Thessaloniki, Greece; ^6^ Cluster of Excellence Physics of Life, Technische Universität Dresden, 01307 Dresden, Germany

**Keywords:** Hypoxia, Hif1a, WNT, Gastruloid, Pluripotency

## Abstract

The cellular microenvironment, together with intrinsic regulators, shapes stem cell identity and differentiation capacity. Mammalian early embryos are exposed to hypoxia *in vivo* and appear to benefit from hypoxic culture *in vitro*. Yet, how hypoxia influences stem cell transcriptional networks and lineage choices remain poorly understood. Here, we investigated the molecular effects of acute and prolonged hypoxia on embryonic and extra-embryonic stem cells as well as the functional impact on differentiation potential. We find a temporal and cell type-specific transcriptional response including an early primitive streak signature in hypoxic embryonic stem cells mediated by HIF1α. Using a 3D gastruloid differentiation model, we show that hypoxia-induced T expression enables symmetry breaking and axial elongation in the absence of exogenous WNT activation. When combined with exogenous WNT activation, hypoxia enhances lineage representation in gastruloids, as demonstrated by highly enriched signatures of gut endoderm, notochord, neuromesodermal progenitors and somites. Our findings directly link the microenvironment to stem cell function and provide a rationale supportive of applying physiological conditions in models of embryo development.

## Teaser

**Summary:** Prolonged hypoxia (reduced oxygen tension) induces WNT pathway activity and is harnessed to enhance differentiation of embryonic stem cells into gastruloids.

## INTRODUCTION

In most early mammalian embryos, the first three cell types are established and maintained in oxygen tension ranging from 1.5 to 8% (Fischer and Bavister, 1993; Ottosen et al., 2006; Yedwab et al., 1976). These comprise the pluripotent epiblast, the primitive endoderm (PrE), and the trophectoderm (TE). Upon differentiation, the extra-embryonic PrE and TE give rise to the yolk sac and placenta, respectively (Rossant et al., 2003). Local oxygen levels in the embryo are likely sub-atmospheric until proper placentation at midgestation (Woods et al., 2018). Hypoxia is also endemic to adult stem cell niches (Mohyeldin et al., 2010) and solid tumors (Muz et al., 2015; Rankin and Giaccia, 2016) and as such is a common physiological component of the cellular microenvironment.

Numerous studies on mouse and human embryonic stem cells (ESCs) revealed that hypoxia promotes ESC differentiation, especially towards endodermal lineages (Burr et al., 2017; Forristal et al., 2010; Houghton, 2021; Kusuma et al., 2014; Pimton et al., 2015). Hypoxia is also implemented in some protocols that model mammalian embryo development in a dish (Sozen et al., 2021; Aguilera-Castrejon et al., 2021), yet the mechanisms through which hypoxia exerts its beneficial effects are not clear. The severity and duration of hypoxia are major determinants of the hypoxic response, which is primarily executed by hypoxia-inducible factors (Hif). The canonical hypoxic response entails activation of glycolysis and angiogenesis genes by HIF1α (Muz et al., 2015; Podkalicka et al., 2020). In addition, hypoxia contributes to epithelial-mesenchymal transition (EMT) and invasiveness in various cancers (Muz et al., 2015; Rankin and Giaccia, 2016). EMT is also a cornerstone of embryonic development, as it enables cell movement and migration. In the mouse embryo, radial symmetry breaking happens at the time of gastrulation via formation of the primitive streak (Bardot and Hadjantonakis, 2020). The interplay between signaling pathways, including WNT, BMP and FGF, and downstream activities of master transcription factors (TFs), such as Eomes, T (brachyury) and Snai1 induce EMT on the posterior side of the embryo (Bardot and Hadjantonakis, 2020). Antagonization of these signals then defines the ectoderm on the anterior side (Bardot and Hadjantonakis, 2020).

Although WNT is not required for mouse pre-implantation development (Fan et al., 2020), distinct WNT activities mediate long-term maintenance (ten Berge et al., 2011; Fan et al., 2020) and resolution of pluripotency (Bardot and Hadjantonakis, 2020) *in vivo*. *In vitro*, distinct pluripotent states can be captured (Kinoshita et al., 2021; Neagu et al., 2020; Ying et al., 2008) and differentiation programs can be mimicked (van den Brink et al., 2014; Hayashi et al., 2011) by modulating the activity of developmental signaling pathways including the WNT pathway. Exogenous WNT activation during differentiation of ESC aggregates of a defined size leads to *T* expression and axial elongation, thus generating embryonic organoids resembling the post-implantation embryo (gastruloids) (Beccari et al., 2018; van den Brink et al., 2014; Turner et al., 2017; Vianello and Lutolf, 2020 preprint). Prolific WNT activity is, however, undesirable as it hinders the emergence of certain tissues, including the neural lineages and reduces structural complexity (Girgin et al., 2021; Olmsted and Paluh, 2021). *In vivo*, transient and fine-tuned gene activity underlies tissue diversification in the gastrulating embryo (Mittnenzweig et al., 2021). Modulation of pathway activity is thus crucial to achieve *in vivo*-like complexity in models of embryo development.

Here, we investigated the molecular effects of acute and prolonged hypoxia on transcriptional networks and stem cell identities of embryonic and extra-embryonic stem cells. We show that hypoxic ESCs are transcriptionally primed with a primitive streak signature, including *Wnt3* and *T* induction downstream of HIF1α. Exposure to hypoxia enables spontaneous symmetry breaking in gastruloids without exogenous WNT activation. Conventional gastruloids generated via ubiquitous WNT activation also benefit from hypoxia and present an enhanced variety of cell states compared with normoxic gastruloids, namely gut endoderm, notochord, as well as mesodermal progenitors and derivatives.

## RESULTS

### Exposure of stem cells to hypoxia leads to cell type-specific transcriptional responses

**Fig. 1.**
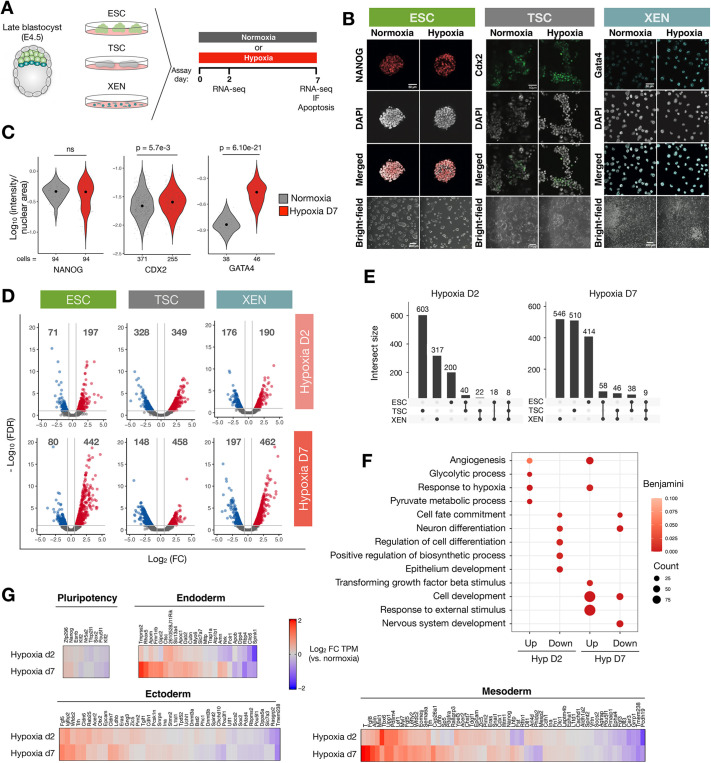
**Hypoxia elicits cell type-specific transcriptional responses in ESCs, TSCs and XEN cells.** (A) Schematic of the experimental setup. IF, immunofluorescence. (B) Immunofluorescence staining and brightfield images of ESCs, TSCs and XEN cells cultured in normoxia or hypoxia for 7 days. Scale bars: 50 μm (immunofluorescence); 200 μm (brightfield). (C) Quantification of immunofluorescence images including those displayed in B. The fluorescent intensity of each nucleus was measured and normalized to the nuclear area. Each fine dot represents a cell. *n* represents the number of quantified cells per sample. Statistical tests are unpaired, two-sample Wilcoxon tests. (D) Volcano plots showing upregulated (red) and downregulated (blue) genes on hypoxia day 2 and day 7 relative to normoxia. Numbers of DE genes are indicated above each section. (E) Overlap of DE genes across cell types on hypoxia day 2 and day 7. Schematics below graphs indicate the samples in which the genes were identified as DE. (F) GO-BP terms associated with DE genes in ESCs exposed to acute (d2) or prolonged (d7) hypoxia (Hyp). Representative significant terms are shown. (G) Heatmaps showing expression levels of the indicated pluripotency and lineage-associated genes in hypoxic relative to normoxic ESCs. See Materials and Methods for details.

To investigate the impact of hypoxia on transcriptional programs of mouse early embryonic and extra-embryonic stem cells, we used ESCs (Evans and Kaufman, 1981; Martin, 1981), extra-embryonic endoderm stem (XEN) cells (Kunath et al., 2005) and trophoblast stem cells (TSCs) (Tanaka et al., 1998), which are *in vitro* models of the epiblast, PrE and TE, respectively. The acute response to hypoxia in the form of Hif activation and metabolic adjustments occurs within minutes to hours (Pagé et al., 2002). However, the embryo might be exposed to hypoxia for about 9-10 days *in vivo* between fertilization and approximately embryonic day (E) 9-10 when proper placentation begins (Woods et al., 2018). Early embryonic and extra-embryonic cells are thus likely exposed to hypoxia for at least a few days. To examine the effect of hypoxia on stem cell identity and function in a time-resolved manner, we cultured ESCs, TSCs and XEN cells in hypoxia (2% O_2_) or normoxia (20% O_2_) for up to 7 days and assessed stem cell marker expression and proliferation and apoptosis rates (Fig. 1A). In general, prolonged culture in hypoxia did not lead to increased cell death or morphological changes (Fig. S1A, Fig. 1B). ESCs proliferated slower in hypoxia (*P*=0.01), but showed no alterations in cell cycle distribution (Fig. S1B,C). TSC and XEN proliferation was largely not affected (Fig. S1B). Importantly, stem cells remained undifferentiated and retained markers associated with each stem cell state, such as NANOG, CDX2 and GATA4, after 7 days of hypoxia exposure with even higher expression of CDX2 and GATA4 in hypoxia (Fig. 1B,C). Thus, stem cells are adaptable to hypoxia without compromise to their cell state.

To probe the extent and specificity of the transcriptional response to hypoxia, we next profiled gene expression in each stem cell type on day 2 (acute) or day 7 (prolonged) of hypoxia exposure by bulk RNA sequencing (RNA-seq). Cells were cultured at similarly low densities across conditions to eliminate colony size as a barrier to oxygen diffusion (Fig. S1D). Hierarchical clustering based on transcriptome profiles revealed primarily cell type-mediated clusters, indicating that hypoxia in general does not perturb transcriptional identities (Fig. S1E,F). In line with this observation, a moderate number of genes was significantly differentially expressed (DE) in each cell type and time point (ESCs: 268 and 522 genes; TSCs: 677 and 606 genes; XEN cells: 366 and 659 genes on day 2 and day 7, respectively) (Fig. 1D, Table S1). DE genes were largely cell type specific with minimal overlap (Fig. 1E). Gene ontology analysis showed that the early response to hypoxia entails upregulation of glycolysis and angiogenesis genes in ESCs, but not TSCs and XEN cells (Fig. 1F, Fig. S1G,H, Table S2). In all cell types, genes associated with cell development and differentiation were more deregulated on day 7, indicating a temporally progressive transcriptional response to hypoxia (Fig. 1F, Fig. S1G,H). In ESCs, early mesoderm and endoderm-instructive genes, such as *T*, *Tbx6*, *Fgf8* and *Tmprss2*, were selectively upregulated in hypoxia (2- to 4-fold compared with normoxia), whereas early ectoderm and pluripotency genes showed a less dynamic response with little temporal progression (Fig. 1G). Genes that are later associated with node, notochord and primitive gut development (Grosswendt et al., 2020), such as *Krt19*, *Krt7* and *Mixl1*, were also mostly upregulated, whereas those associated with brain and spinal cord development showed a mixed response (Fig. S1I). Thus, key mesoderm and endoderm lineage regulators appear to be specifically induced in hypoxic ESCs. Importantly, even if these gene expression changes do not lead to spontaneous differentiation, they may affect developmental trajectories later. We hereafter focus on ESCs to investigate the hypoxia-associated activity of lineage-specific genes and its functional implications.

### WNT pathway genes are gradually upregulated during prolonged hypoxia

**Fig. 2.**
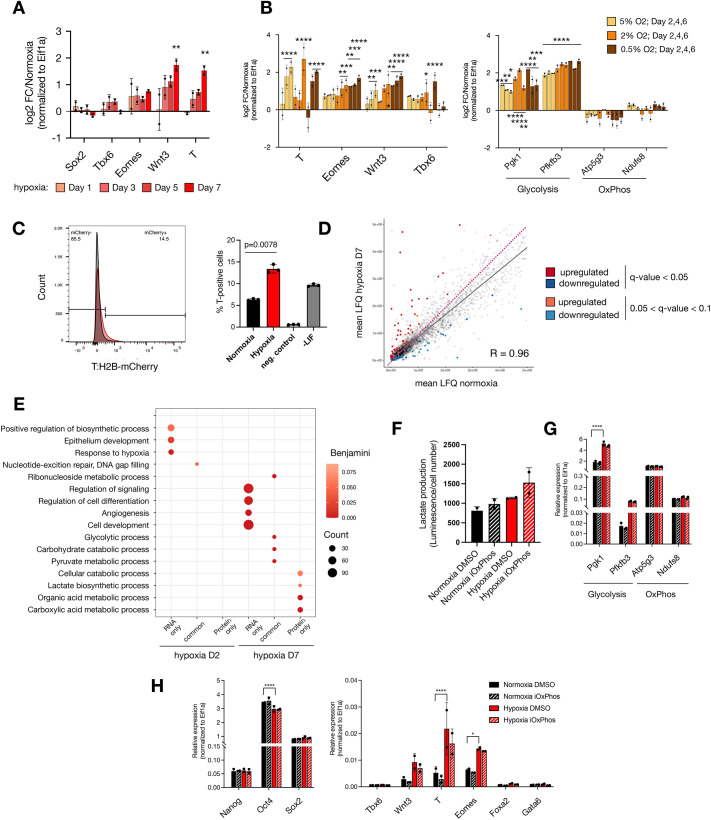
**Transcriptional priming of ESCs in hypoxia by induction of WNT pathway genes.** (A) RT-qPCR analysis of the indicated genes in hypoxic ESCs. (B) Expression levels of the indicated genes under different oxygen tensions. Statistical test was two-way ANOVA for A,B. (C) Expression levels of the T::H2B-mCherry reporter (Veenvliet et al., 2020) in normoxic versus hypoxic cells as determined by flow cytometry. Representative flow cytometry readouts (left) and number of T-positive cells within the cell population are shown. Three biological replicates were performed. Negative control cells were unstained wild-type ESCs. Positive control was differentiation mediated by LIF withdrawal for 7 days (−LIF). Statistical test was two-tailed, paired Student's *t*-test. (D) Proteome profiles of hypoxic (d7) versus normoxic ESCs. Pink dotted line depicts the diagonal. (E) GO terms associated with identified DE transcripts and proteins in hypoxia. Representative significant terms are shown. (F) Measurement of the lactate produced by hypoxic and normoxic ESCs treated with DMSO or iOxPhos. Two biological replicates are shown. Statistical test was one-way ANOVA; all comparisons show non-significant differences. (G) Relative expression levels of selected genes in hypoxic and normoxic ESCs treated for 7 days with DMSO or iOxPhos. Statistical test was two-way ANOVA. (H) Relative expression levels of selected pluripotency- (left) and lineage- (right) associated genes in the indicated conditions. Statistical test was two-way ANOVA. Error bars represent s.d.

The induction of *T*, *Eomes*, *Tbx6* and *Lef1*, among other genes, points towards increased WNT pathway activity in hypoxia (Fig. 1G, Fig. S2A), which was previously observed in cancer cells and linked to EMT (Muz et al., 2015; Rankin and Giaccia, 2016). Of all Wnt genes, *Wnt3*, *Wnt4*, *Wnt7a* and *Wnt7b* were significantly upregulated [fold change (FC)>1.5, false discovery rate (FDR)≤0.1] in hypoxic ESCs (Fig. S2B). WNT3 activity in the posterior primitive streak mediates EMT during gastrulation by upregulating downstream targets such as *T*, *Eomes* and *Tbx6* (Bardot and Hadjantonakis, 2020). To investigate the temporal dynamics of the WNT pathway activity and its relation to oxygen availability, we next cultured ESCs in normoxia or varying degrees of hypoxia for up to 7 days and collected samples at regular intervals for qRT-PCR (Fig. 2A,B). *Wnt3* and *T* were gradually upregulated in hypoxia with an up to ∼4-fold increase over normoxia by day 7, whereas *Eomes* and *Tbx6* were more modestly upregulated (∼1.5-fold, Fig. 2A). Pluripotency and endoderm markers remained largely unchanged (Fig. S2C). Importantly, *Wnt3* and *Eomes* levels were inversely correlated with oxygen availability, indicating association with an oxygen-mediated process (Fig. 2B). Expression of pluripotency markers or oxidative phosphorylation genes did not correlate with oxygen levels, whereas glycolysis genes were upregulated in more severe hypoxic conditions (Fig. 2B, Fig. S2D). *Wnt3*, *T* and *Eomes* induction persisted and further increased at least until day 16 of prolonged hypoxia (Fig. S2E). Return of cells to normoxia restored original expression levels in normoxia, indicating the reversibility of the WNT pathway induction (Fig. S2E). Glycolysis genes were expressed at similar levels throughout the treatment, indicating that the hypoxia response is continuously in place (Fig. S2F). These results further corroborate the hypoxia-mediated selective induction of a transcriptional primitive streak signature that includes WNT pathway genes in ESCs. To understand the heterogeneity of *T* expression in the ESC population, we analyzed an ESC line carrying a T:H2B-mCherry reporter, in which transcriptional activation of *T* is read out as histone H2B coupled to mCherry by flow cytometry (Veenvliet et al., 2020). On day 7 of hypoxia, mCherry was detected in 13-15% of ESCs, compared with baseline expression of ∼6% in normoxic ESCs (Fig. 2C). Thus, *T* is upregulated in a subpopulation of ESCs in response to hypoxia.

Oxygen depletion leads to curtailing of energy-dense processes, including protein synthesis (Koumenis et al., 2002; Lee et al., 2020; Pettersen et al., 1986). To investigate whether differential expression at the transcript level is reflected at the protein level, we performed label-free mass spectrometry on normoxic and hypoxic ESCs (Fig. 2D). Mass spectrometry identified 4260 proteins (Table S3). In general, hypoxic ESCs retained a similar proteome composition, but on average fewer copy numbers per protein compared with normoxic ESCs on day 7 (Fig. 2D). On day 7 in hypoxic ESCs, 63 and 43 proteins were significantly differentially up- or downregulated, respectively (Fig. 2D, Table S4; FC>1.5, q-value<0.05, Spearman R=0.96). DE proteins on day 7 were enriched for metabolic functions (Fig. 2E, Tables S4, S5). In contrast, development- and differentiation-associated genes were DE mostly at the transcript but not protein level (Fig. 2E). This may be due to the limited sensitivity of mass spectrometry or alternatively due to selective translation or post-translational degradation of specific gene products. Western blotting of whole-cell extracts showed low level but gradually increasing expression of T and EOMES in hypoxic ESCs, whereas WNT3 could not be detected (Fig. S2G).

To assess whether T is functional under these conditions in ESCs, we analyzed the expression levels of T target genes (those bound and regulated by T during *in vitro* primitive streak differentiation; Lolas et al., 2014) in hypoxic ESCs (Fig. S2H). Several DE genes were found among putative T target genes but these constitute a small fraction of the total identified (4.25% of T target genes were DE). Furthermore, almost an equal number of putative T target genes were upregulated on day 2 and day 7. Given that T induction at day 2 was modest (∼1.2-fold) at the transcript and protein level (Fig. S2A,G), it is unlikely to be sufficient to directly activate downstream genes at that stage. In addition, putative T-activated genes were present among hypoxia-downregulated as well as hypoxia-upregulated genes (Fig. S2H). We deduce that in ESCs hypoxia induces a transcriptional early primitive streak signature, which is not directly reflected at the protein level. As such, hypoxic ESCs do not resemble epiblast stem cells derived from post-implantation embryos, which comprise a mixture of T-, FOXA2- and SOX2-expressing cells with anterior primitive streak characteristics (Kojima et al., 2014; Tsakiridis et al., 2014).

### Altering lactate production does not replicate the hypoxia-mediated early primitive streak signature

A major component of the hypoxia response is the shift of cellular metabolism from oxidative phosphorylation to glycolysis. Indeed, metabolic rewiring constitutes the main hypoxia response in ESCs at the protein level (Fig. 2E). Metabolic pathways not only determine ways of energy utilization, but also impact cellular states and developmental phenomena (Bulusu et al., 2017; Hu et al., 2020; Oginuma et al., 2017; Rodriguez–Terrones et al., 2020). Therefore, we next set out to dissect the influence of lactate production from other hypoxia-mediated events in the induction of early primitive streak genes. For this, we treated cells with an inhibitor of oxidative phosphorylation (iOxPhos) (Fig. 2F-H), which led to increased lactate production (Fig. 2F). The expression levels of glycolytic enzymes increased in hypoxia but not upon iOxPhos treatment (Fig. 2F,G). Although leading to increased lactate production, iOxPhos treatment in normoxia or in hypoxia did not by itself induce *Wnt3*, *T* or *Eomes* expression, nor did it affect other developmental or pluripotency-associated genes (Fig. 2H). Overall, these results suggest that the induction of developmental genes in hypoxia cannot be replicated only by altering the glycolytic output.

### Chemical activation of HIF1α replicates WNT pathway induction by hypoxia

**Fig. 3.**
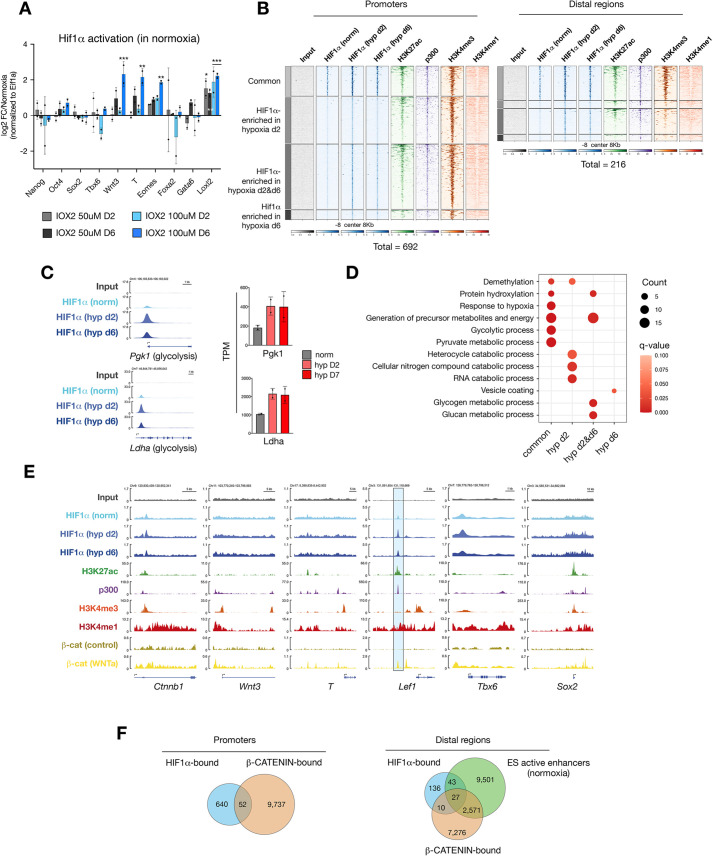
**HIF1α mediates induction of WNT pathway genes in ESCs.** (A) RT-qPCR analysis of the indicated genes in normoxic ESCs treated with the HIF1α activator IOX2 at the indicated concentrations for 2 or 6 days. Data represent log_2_FC over DMSO-treated ESCs. Statistical test was two-way ANOVA. (B) Density plots showing enrichments of the indicated genes and histone modifications at HIF1α-bound promoters and distal regions. ±8 kb surrounding peak center is shown. Numbers below the density plots indicate the total number of HIF1α-bound sites on each day. (C) Left: Genome browser views of HIF1α binding at the promoters of the indicated glycolysis genes. Right: Expression values of the indicated genes in normoxic and hypoxic ESCs as measured by RNA-seq. (D) GO-BP terms associated with HIF1α-target genes with HIF1α binding at promoters. Representative significant terms are shown. (E) Genome browser views of chromatin occupancy at canonical WNT pathway genes. Blue highlight depicts an active enhancer in ESCs. (F) Venn diagrams showing the numbers of overlapping peaks with HIF1α and β-catenin potential co-binding at promoters (left) and ESC active enhancers (right). Error bars represent s.d. in A,C.

We next probed the primary hypoxia effector transcription factor HIF1α for its potential regulation of early primitive streak genes. To test whether HIF1α activation is sufficient to upregulate these genes, we treated ESCs with the small molecule IOX2, which inhibits the HIF1α destabilizer prolyl hydroxylase 2, thus leading to HIF1α stabilization and consequent activation of its target genes in a dose-dependent manner in normoxia (Fig. S3A) (Chowdhury et al., 2013; Murray et al., 2010). IOX2 treatment thus allows the HIF1α-mediated response to be distinguished from possible other components of hypoxia. Treatment of ESCs with IOX2 resulted in the induction of *Wnt3*, *T* and *Eomes* in a dose-dependent manner and at levels comparable to hypoxia (Fig. 3A). Similar to hypoxia, these genes were induced after prolonged treatment, whereas glycolysis genes were upregulated as early as day 2 (Fig. 3A, Fig. S3A).

We then investigated whether developmental genes are induced by direct HIF1α binding at promoters by profiling the genomic occupancy of HIF1α (Fig. 3B). HIF1α was found to occupy the promoters of 141, 648 and 478 genes in normoxia, and on days 2 and 6 of hypoxia, respectively (Table S6). Of these peaks,137 were common to all conditions and 297 were shared in hypoxia days 2 and 6 (Fig. S3B). In general, HIF1α appears to mainly directly regulate metabolic genes and neither temporally nor conditionally binds developmental gene promoters (Fig. 3C-E, Table S7). In particular, we did not detect HIF1α binding at promoters of WNT pathway effectors except for β-catenin, which, however, did not result in altered expression (Fig. 3E, Fig. S3C,D, Table S1). Furthermore, only 36 out of 478 HIF1α-bound genes were upregulated on day 6 of hypoxia exposure. These results suggest that HIF1α mediates the induction of early primitive streak genes in hypoxia, but not via direct promoter binding.

In addition to promoters, HIF1α also binds 216 distal regions, 70 of which are enriched for H3K27 acetylation, H3K4me1 and p300 and are devoid of H3K4me3 in line with an enhancer signature (Fig. 3B, Fig. S3B, Table S6) (Cruz-Molina et al., 2017). We found HIF1α binding at regions proximal to several developmental genes, including the WNT effector transcription factor *Lef1*, which is upregulated on day 2 of hypoxia (Fig. 3E, Fig. S3D). The *Lef1* distal region is bound by β-catenin in WNT-stimulated ESCs but not in normal ESC culture conditions [Fig. 3E, compare β-catenin (WNTa) versus β-catenin (control); data from Zhang et al., 2013]. Although we did not observe β-catenin stabilization or mainly nuclear localization in hypoxia as reported previously (Mazumdar et al., 2010) (Fig. S3C), we did find that HIF1α localization overlapped with the distal β-catenin-binding site, suggesting potential colocalization (Fig. 3E). More generally, HIF1α and β-catenin shared 52 target promoters and 27 target ESC active enhancers (Fig. 3F). Thus, a HIF1α/β-catenin axis may selectively induce developmental genes in hypoxia. Interestingly, HIF1α also bound several chromatin regulators in normoxia as well as hypoxia (Fig. S3E), suggesting that it may alter epigenetic landscapes in hypoxia.

### Hypoxic gastruloids spontaneously elongate in the absence of exogenous WNT activation

**Fig. 4.**
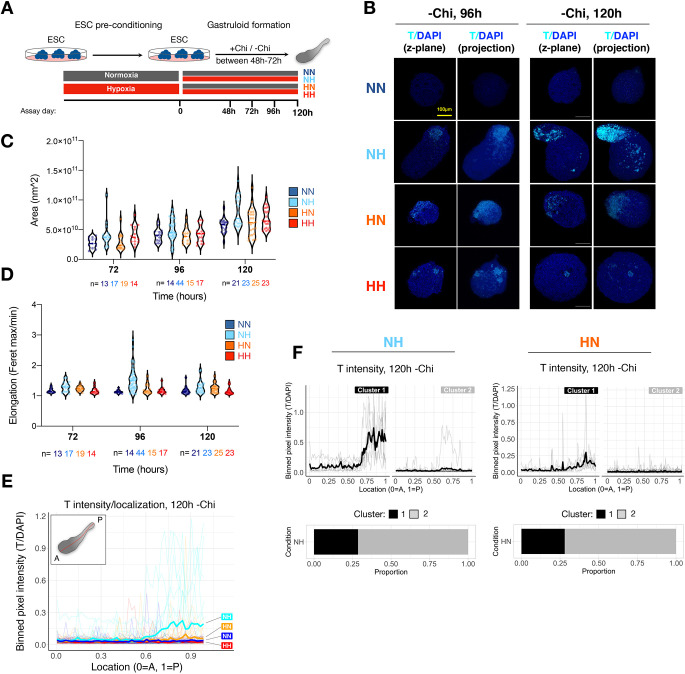
**Hypoxia can induce spontaneous elongation of gastruloids in the absence of exogenous WNT activation.** (A) Schematic of the experimental setup. The number of structures used for each time point and condition can be found in Table S9. (B) Confocal fluorescence microscopy images of representative −Chi gastruloids at 96 h and 120 h of culture (‘projection’ indicates three-dimensional projection). Scale bar: 100 μm. (C) Area of gastruloids at each time point and condition. Fluorescent images were used for quantification. Each dot indicates a single structure and *n* indicates the number of analyzed structures at each time point and condition. (D) Elongation index (defined as aspect ratio Feret max/Feret min) of gastruloids at each time point and condition. (E) Localization of T signal along the A-P axis of gastruloids (as shown in inset) in each condition at 120 h of culture. T signal was normalized to DAPI and binned at 1% length increments along each structure for plotting. See Materials and Methods for details. Thick lines show mean values and thin lines show data from individual structures. (F) k-means clustering of the NH and HN structures presented in E with *n*=2 clusters. Experimental conditions: HH, hypoxia-hypoxia; HN, hypoxia-normoxia; NH, normoxia-hypoxia; NN, normoxia-normoxia.

To test functionally whether the hypoxia-induced early primitive streak signature enables the emergence of cell states and tissues that resemble and arise from the primitive streak *in vivo*, we employed the gastruloid model. In this model, 3D aggregates are generated from defined, low numbers of ESCs (∼200-400), and then transiently treated with an exogenous WNT activator [here CHIR99021 (Chi) is used] between 48 h and 72 h of culture (Fig. 4A). Under these conditions, aggregates undergo symmetry breaking, elongation and self-organization of the body axes, and show polarized T expression at the posterior end (Beccari et al., 2018; van den Brink et al., 2014; Turner et al., 2017). Concomitant with axial elongation and WNT/T activation, derivatives of the three germ layers emerge (Beccari et al., 2018; van den Brink et al., 2014; Turner et al., 2017; Vianello and Lutolf, 2020 preprint). Without exogenous WNT activation, ESC aggregates remain as embryoid bodies and rarely spontaneously elongate. We hypothesized that the hypoxia-mediated induction of a transcriptional early primitive streak signature might suffice to enable spontaneous symmetry breaking and axial elongation in the absence of exogenous WNT activation (termed ‘−Chi’ hereafter). To test this possibility, we generated aggregates from 400 ESCs in normoxia or hypoxia and collected them for analysis at 48, 72, 96 and 120 h post-aggregation (Fig. 4A, Fig. S4A). Structures were stained for T and with a DNA-binding dye (DAPI) and imaged using confocal microscopy (Fig. 4B, Fig. S4B). Size and elongation index were calculated using DAPI as counterstain (Fig. 4C,D). Whereas structures cultured in normoxia throughout the procedure did not express T or spontaneously elongate, pre-conditioning of ESCs in hypoxia prior to aggregation (HN) induced T expression and slight elongation [Fig. 4B-D, Fig. S4A,B, compare HN to NN (normoxia-normoxia) condition at 120 h]. Strikingly, exposure to hypoxia only during differentiation (NH condition) led to stronger localized T expression and more pronounced spontaneous elongation in a subset of structures (Fig. 4B-D, Fig. S4A,B). Hypoxia-induced spontaneous elongation was WNT pathway dependent, as treatment with the PORCN inhibitor LGK-974 and the tankyrase inhibitor XAV939 during hypoxia exposure resulted in loss of spontaneous elongation and induction of endogenous *T* and *Eomes* as well as the T::H2B-mCherry reporter (Figs S4C, S5A-C, Movie 1).

To probe T expression dynamics and variability in −Chi structures further, we performed quantitative image analysis of 90 structures collected at 96 h and 92 structures collected at 120 h post-aggregation in four independent experiments (Fig. 4E,F, Fig. S4D,E; see Table S9 for number of structures used for all experiments). T signal was binned along the anterior-posterior (A-P) axis and normalized to DAPI (Fig. 4E). This analysis showed T induction in all hypoxic conditions at 96 h post-aggregation, which, however, was only clearly detected in the NH −Chi condition at 120 h post-aggregation (Fig. 4E, Fig. S4D). k-means clustering of T expression patterns showed that a T-positive pole occurs at about the same rate in NH and HN −Chi aggregates (∼30% of all structures in NH or HN versus 0% in the NN condition) (Fig. 4F, Fig. S4E). However, T expression level was lower in HN −Chi structures compared with NH −Chi (cluster 1 in each condition, Fig. 4F), which might explain the better elongation of NH −Chi structures at 120 h (Fig. 4D). HH −Chi structures neither showed a T-positive pole nor elongated, despite showing T expression at 48 and 72 h, pointing to precise level and temporal dynamics of T expression as critical parameters of spontaneous elongation (Fig. 4D-F, Fig. S4B). These findings suggest that exposure to hypoxia equips ESC aggregates with the capacity to self-initiate the developmental events characteristic of the post-implantation embryo, including symmetry breaking, polarization and axial elongation, pointing to the importance of the microenvironment in shaping cell commitment and tissue morphogenesis.

### Hypoxia modulates T expression dynamics in gastruloids

**Fig. 5.**
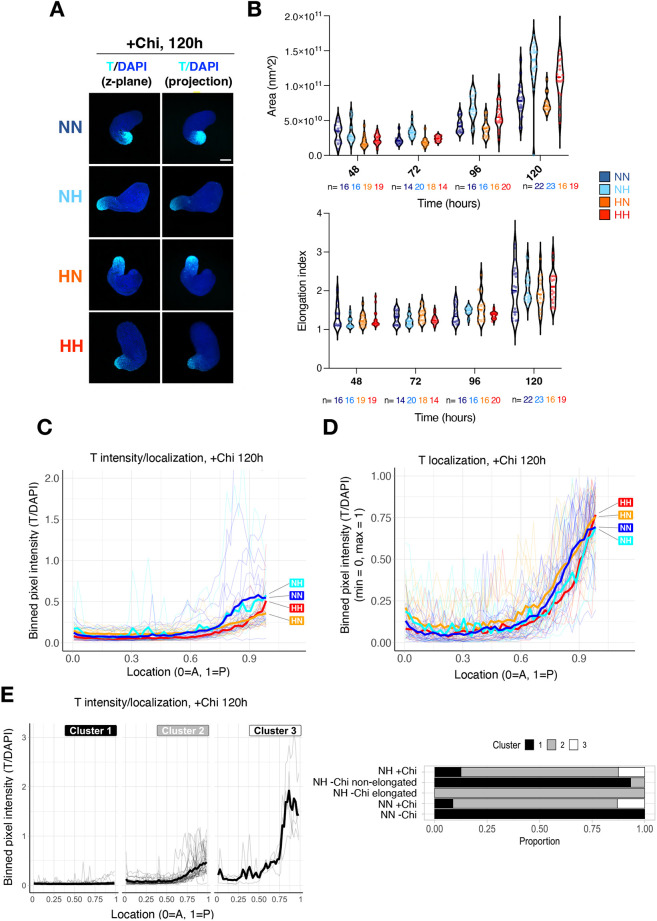
**Hypoxia modulates T levels in conventional (+Chi) gastruloids.** (A) Confocal fluorescence microscopy images of representative +Chi gastruloids at 120 h of culture. The number of structures used for each experiment is available in Table S9. (B) Area and elongation index (defined as aspect ratio Feret max/Feret min) of +Chi gastruloids at each time point and condition. Each dot indicates a single structure and *n* indicates the number of analyzed structures at each time point and condition. (C,D) Localization of T signal along the A-P axis of gastruloids in each condition. T signal was binned at 1% length increments along each structure for plotting. T intensity was normalized to DAPI stain, then was either plotted as such (C) or was further fitted in a 0-1 scale (D). Thick lines show the mean and thin lines show data from individual structures. (E) k-means clustering of the indicated conditions at 120 h with *n*=3 clusters. Experimental conditions: HH, hypoxia-hypoxia; HN, hypoxia-normoxia; NH, normoxia-hypoxia; NN, normoxia-normoxia.

Although −Chi HN and, in particular, NH gastruloids can spontaneously break symmetry, polarize and elongate, gastruloid formation efficiency and elongation capacity is limited under these conditions. To investigate hypoxia-induced T activity in a more robust model of embryo development, we next combined hypoxia with exogenous activation of WNT (termed ‘+Chi’ hereafter). In general, most +Chi structures elongated and showed posterior T localization by 120 h after aggregation (Fig. 5A,B, Fig. S6A,B). Performing aggregation in hypoxia resulted in larger gastruloids (Fig. 5B). Quantitative, spatially resolved analysis of T expression along the A-P axes of +Chi structures revealed that hypoxia modulates T expression levels and patterns. At 96 h post-aggregation, NH +Chi gastruloids showed lower and more posterior-localized T expression compared with conventional NN +Chi gastruloids (Fig. S6C,D). HN and HH gastruloids expressed T at similar levels to NN gastruloids at the posterior pole but lower at more anterior regions, indicating that T is on average more confined to the posterior end (Fig. S6C,D). At 120 h, T was confined to the posterior end in all conditions, with HN and HH gastruloids showing on average lower T expression (Fig. 5C,D).

Intriguingly, direct comparison of T localization and intensity in +Chi and −Chi gastruloids revealed that in the subset of −Chi NH gastruloids that express T at 120 h, T levels and localization were similar to those observed in +Chi gastruloids at 120 h (Fig. 5E). In contrast, hyper-induction of T was seen in ∼15% of +Chi gastruloids but in none of the −Chi NH gastruloids (Fig. 5E). Although T expression was similarly confined to the posterior end across conditions and in +Chi and −Chi experiments, its expression dynamics were distinct at earlier time points. Whereas in −Chi gastruloids T was spontaneously induced in a small cluster of cells and already confined to the posterior end at 72 h, in +Chi gastruloids T was initially induced in most cells by 72 h and later confined to the posterior end at 96 h (Fig. S6B). Taken together, the mode of induction and expression dynamics of T is modulated under hypoxia and these distinct patterns might influence differentiation trajectories in gastruloids.

### Enhanced representation of germ layer markers in conventional gastruloids cultured in hypoxia

**Fig. 6.**
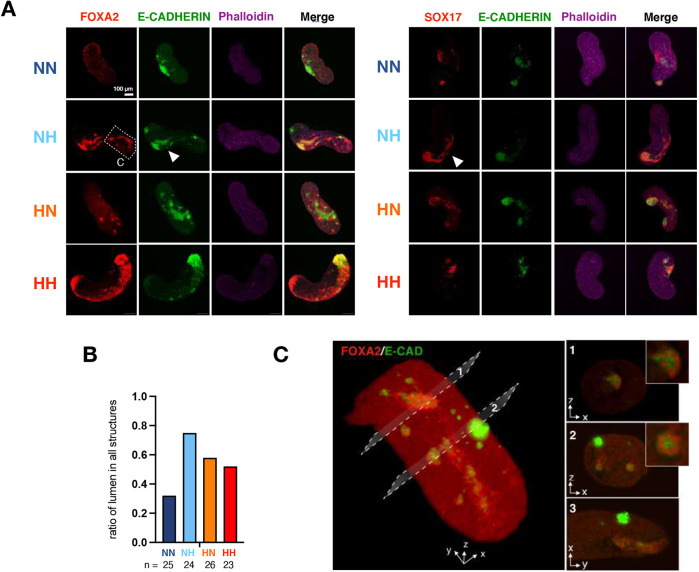
**Hypoxic +Chi gastruloids show enhanced endoderm signatures.** (A) Representative images of FOXA2/E-cadherin (cadherin 1)/Phalloidin (left) or SOX17/E-cadherin//Phalloidin (right) staining in normoxic and hypoxic +Chi gastruloids. Scale bar: 100 µm. Arrowheads indicate gut tube-like organized structures. (B) The ratio of structures containing a lumen surrounded by FOXA2- or SOX17-positive cells. *n* indicates number of analyzed structures. (C) Light-sheet microscopy images of a +Chi NH gastruloid at 120 h. A 3D reconstruction (left) and cross-sections (right) are shown. Insets on the right show the gut tube-like structures in more detail. The image captures the area marked by the dashed box in A. Experimental conditions: HH, hypoxia-hypoxia; HN, hypoxia-normoxia; NH, normoxia-hypoxia; NN, normoxia-normoxia.

To test whether hypoxic gastruloids harbor more diverse cell types and cellular structures compared with conventional normoxic gastruloids, possibly due to modulation of T levels, we first analyzed the expression patterns of key endoderm lineage markers, such as FOXA2 and SOX17 (Fig. 6A). In conventional NN +Chi gastruloids, FOXA2 and SOX17 expression remained minimal and a primitive lumen opening could be found in only ∼30% of structures (Fig. 6A,B). In contrast, expression of endoderm markers was remarkably enhanced in hypoxic gastruloids (Fig. 6A). Furthermore, these endodermal cells appeared to frequently self-organize around lumen openings reminiscent of the embryonic gut tube (Fig. 6A, arrowheads). To investigate these potential gut tube-like structures in more detail, we performed light-sheet microscopy and also inspected E-cadherin, a marker of polarized epithelium, in relation to FOXA2-expressing cells (Fig. 6C). Light-sheet microscopy imaging clearly showed epithelial polarization and rudimentary lumen openings of gut tube-like structures in NH +Chi gastruloids at 120 h (Fig. 6C).

We next investigated the abundance of neuro-ectodermal cells in hypoxic gastruloids by staining for SOX2 and SOX1 (Fig. S7A,B). In NN +Chi gastruloids, SOX2 expression pattern was highly variable and either covered the entire A-P axis or remained localized to the posterior end (Fig. S7A). In hypoxic NH and HN gastruloids, we observed on average an expansion of the SOX2-expressing domain (Fig. S7A). In contrast, the more advanced neuroectoderm marker SOX1 was less abundant in hypoxia (Fig. S7B). Levels of the pluripotency-associated protein OCT4 (POU5F1) remained similar in +Chi gastruloids at 120 h; therefore, the higher number of SOX2^+^ cells likely does not stem from an increase of undifferentiated pluripotent stem cells (Fig. S7B). These results suggest that the neural lineage may be less differentiated in hypoxic +Chi gastruloids and potentially harbor more progenitor cells. Our data collectively point to hypoxia as an important factor in shaping lineage trajectories; thus, we next explored the cell-type composition of hypoxic gastruloids in detail.

### Single-cell RNA-seq reveals enhanced lineage representation in hypoxic gastruloids

**Fig. 7.**
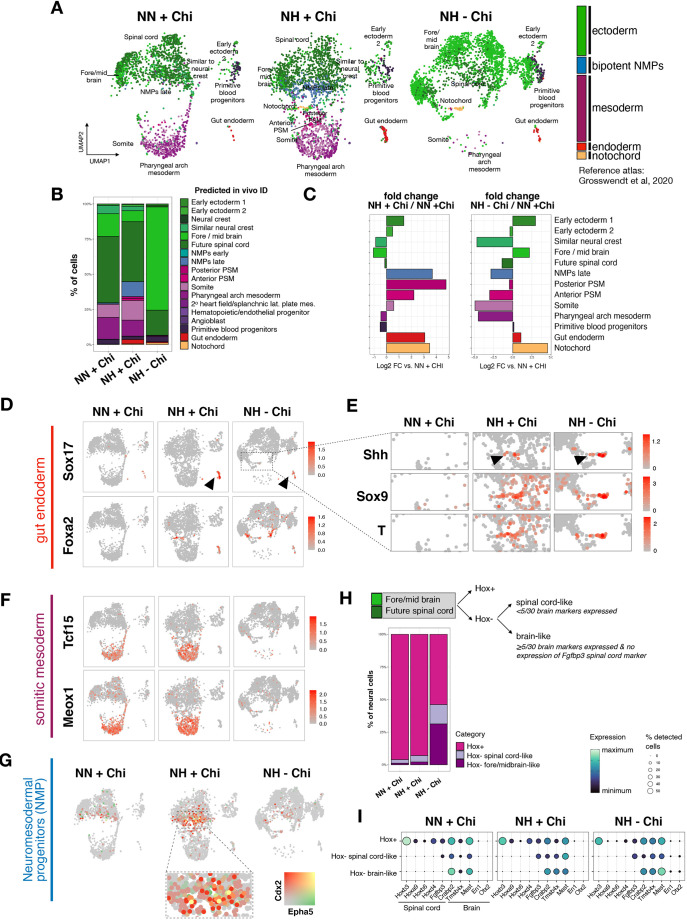
**scRNA-seq reveals enhanced representation of diverse cell types in hypoxic gastruloids.** (A) Uniform manifold approximation and projection (UMAP) with Seurat clusters colored by germ layer according to *in vivo* predicted ID identified by using reference scRNA-seq atlas from E6.5-E8.5 (Grosswendt et al., 2020). Cell states with fewer than five cells are not named on the UMAP. (B) Percentage of cells assigned to the indicated *in vivo* cell states according to reference scRNA-seq atlas. (C) Relative abundance of each cell type in NH +Chi and NH −Chi gastruloids compared with the NN +Chi condition. Cell states with fewer than ten cells across conditions are not shown. (D-G) UMAP feature plots colored by expression of marker genes for gut endoderm (Kanai-Azuma et al., 2002; Wang et al., 2012) (D), notochord (Ang and Rossant, 1994) (E), somitic mesoderm (Grosswendt et al., 2020) (F) and NMPs (Grosswendt et al., 2020; Pijuan-Sala et al., 2019) (G). (H) Top: Schematic overview of neural cell categorization. The annotated forebrain and spinal cord cells from A have been regrouped and reclassified based on the indicated criteria. Bottom: Percentage of cells assigned to the indicated categories. (I) Expression of top genes characterizing the *in vivo* spinal cord and brain cell types. Experimental conditions: HH, hypoxia-hypoxia; HN, hypoxia-normoxia; NH, normoxia-hypoxia; NN, normoxia-normoxia.

To test the impact of hypoxic culture conditions on cellular diversity in detail, we performed single-cell RNA-sequencing (scRNA-seq) analysis of NN +Chi, NH +Chi and NH −Chi gastruloids (Fig. 7). We pooled 28 structures per condition and employed lipid-indices based multiplexing technology (MULTI-seq) to sequence all samples together (McGinnis et al., 2019). After demultiplexing and quality control, we analyzed a total number of 7014 cells (Fig. S8A). Seurat clustering identified ten distinct clusters (Hao et al., 2021) (Fig. 7A). We then annotated these gastruloid cell clusters using two distinct reference scRNA-seq atlases of embryonic stages E6.5-E8.5 (Grosswendt et al., 2020; Pijuan-Sala et al., 2019) (Fig. 7A, Fig. S8B). Coloring of the annotated cell states by germ layer origin revealed a clear increase in endodermal cells in hypoxic gastruloids, both with and without exogenous WNT activation (Fig. 7A, annotated in red). Spontaneously elongating NH −Chi gastruloids showed a substantial increase of neural cells along with a near-complete depletion of mesodermal derivatives, suggesting that spontaneous T induction under hypoxia is not sufficient to generate or sustain mesodermal cells in the absence of exogenous WNT activation (Fig. 7A). Surprisingly, conventional gastruloids when generated in hypoxia (NH +Chi) showed a substantial increase in mesodermal derivatives (Fig. 7A, annotated in purple).

To explore gastruloid cell states further in an unbiased manner, we inspected the predicted *in vivo* cell identity (Fig. 7B, Fig. S8B,C; see Fig. S8D for details on the top *in vivo* marker genes for the distinct predicted *in vivo* cell states). Although in general both reference atlases used for prediction of *in vivo* cell identities generate highly similar results, cell identity assignments are sometimes ambiguous, especially when comparing cell states that are transcriptionally highly similar (Grosswendt et al., 2020; Pijuan-Sala et al., 2019). In these cases, we interrogated known and specific cell-state markers to predict *in vivo* cell IDs.

Calculation of the differential abundance of each cell type over the NN +Chi condition showed a clear enhancement of *Foxa2*- and *Sox17*-expressing gut endodermal cells under hypoxia, confirming our previous observations (Fig. 7C,D). Gut endoderm formation was enhanced with or without exogenous WNT activation (albeit higher in +Chi), pointing to a WNT-independent route to endoderm formation in hypoxic conditions. Interestingly, scRNA-seq also revealed that a cluster of cells with a notochordal transcriptional signature consistently emerges in hypoxic gastruloids independent of Chi treatment (Fig. 7A-C, annotated in orange). These cells co-expressed *T*, *Shh* and *Sox9* and were clearly distinguishable as a distinct population (Fig. 7E). Thus, hypoxia promotes the notochord transcriptional signature in a subset of cells in gastruloids.

Although hypoxic −Chi gastruloids are clearly skewed towards neural identity, in hypoxic +Chi gastruloids we found an increase in presomitic mesoderm (PSM) as well as somitic signatures (Fig. 7C,F, Fig. S8E). This was confirmed in independent samples by quantitative PCR (Fig. S8F). Furthermore, neuromesodermal progenitors (NMPs) were remarkably enriched in NH +Chi gastruloids (Fig. 7G), suggesting that the bipotential capacity of NMPs may generate mesodermal derivatives/somites and neural cells in a more balanced manner when exposed to hypoxia (Fig. 7A,B).

To better understand differences in neural cells, we used the Hox code as a proxy to probe their A-P identity in more detail (Neijts et al., 2014). We regrouped the cells from the fore/midbrain and future spinal code clusters and then assessed Hox gene expression (i.e. Hox expression as a proxy for posterior neural identity that could be associated with the spinal cord or hindbrain) (Fig. 7H). The proportion of Hox-positive posterior cells was decreased slightly in NH +Chi and substantially in NH −Chi gastruloids. Further analysis of the Hox-negative ‘anterior’ cells revealed an increase in both brain-like and spinal cord-like cells (Fig. 7H,I). This analysis suggests that many of the neural cells in NH −Chi gastruloids have a more anterior, brain-like identity, compared with NN or NH +Chi gastruloids, which is, however, likely immature, owing to the lack of *in vivo* mature brain markers such as *Otx2* and *En1* (Fig. 7I).

## DISCUSSION

In this study, we present hypoxia as a crucial microenvironmental factor shaping cell-fate decisions in early stem cells and progeny. We show that (1) endoderm is highly enriched by hypoxia exposure and may form independently of WNT activity via a distinct transcriptional network; (2) a distinct population of node/notochordal cells arise in hypoxia; (3) hypoxia enhances the representation of mesodermal derivatives in conventional gastruloids, likely via more balanced NMP differentiation; and (4) spontaneously elongating gastruloids largely lack mesoderm but contain cells with a more cephalic identity. Taken together, we conclude that hypoxia could be harnessed in the culture of models of embryo development as a means to induce axial elongation and improve germ layer representation. Increased lactate production, a major component of the hypoxia response, does not contribute to the induction of lineage genes in ESCs. Increased glycolysis might, however, contribute to some of the effects detected at the later stages of gastruloid formation. In particular, the increased amount of NMPs and (pre-)somitic mesodermal cells in NH +Chi compared with conventional NN +Chi gastruloids could be related to glycolysis shaping the neuro-mesodermal lineage choice via FGF/WNT pathway activity during axial elongation (Bulusu et al., 2017; Oginuma et al., 2017). Interestingly, the directionality of the effect remains unsettled, with some studies reporting positive regulation of WNT signaling by glycolysis and other studies demonstrating the contrary (Miyazawa et al., 2021 preprint; Oginuma et al., 2017, 2020). Overall, even though the impact of hypoxia at pre-implantation stages remains to be fully resolved, oxygen concentration and metabolic status appear to be stage- and tissue-specific regulators of developmental gene expression and morphology in ESCs and 3D gastruloids.

Direct comparison of −Chi and +Chi gastruloids shows distinct developmental modes underlying T induction, symmetry breaking, polarization and axial elongation. Non-uniform, polarized T induction has been described in two recently published alternative gastruloid protocols, and in both cases leads to more complete models of embryo development that include the cephalic tissues (Girgin et al., 2021; Xu et al., 2021). Based on our findings, hypoxia could be combined with a milder exogenous WNT activation to generate gastruloids that more closely reflect the embryo, and/or harnessed in other models of mouse post-implantation or human development (Moris et al., 2020; Olmsted and Paluh, 2021; Veenvliet et al., 2020; Xu et al., 2021). The potential of such an approach is clearly illustrated by the NH +Chi gastruloids, in which the higher amount of NMPs concomitant with increased expression of PSM and somite markers suggests that hypoxia can be utilized to maintain the bipotential NMPs that contribute cells to both neural tube and somites *in vivo* and shift their decision-making process (Koch et al., 2017; Tzouanacou et al., 2009). In trunk-like structures, about 50% of the structures display a neural bias, with no clear somite formation, whereas in the other 50% formed somites are smaller than their *in vivo* counterparts (Veenvliet and Herrmann, 2021; Veenvliet et al., 2020). It will thus be of great interest to test whether hypoxia can improve trunk-like structure formation. Moreover, the emergence of cells with a node/notochord-like signature in hypoxic gastruloids might be harnessed to improve somite and neural tube patterning in embryo models given the important role of the notochord in the patterning of (par)axial structures (Stemple, 2005; Sagner and Briscoe, 2019). Indeed, an initial analysis of dorsal-ventral patterning signatures of spinal cord cells in our scRNA-seq data revealed increased dorsal and ventral identities along with decreased midline signature in hypoxic gastruloids (Fig. S8G; used marker genes are listed in Table S10). As a result, a dorsal-ventral pattern may emerge, which is in line with the role of notochord-secreted Shh in conferring ventral identity *in vivo* (Sagner and Briscoe, 2019).

Under our culture conditions, hypoxia has a clear endoderm-enhancing effect, in line with what has been reported for spontaneous and directed differentiation of mouse ESCs towards definitive endoderm (Pimton et al., 2015). The efficiency of endoderm induction in conventional gastruloids is currently subject to high variation. Whereas some studies have reported the formation of gut primordia in gastruloids, in other reports mainly neural and mesodermal tissue is formed (Beccari et al., 2018; van den Brink et al., 2020; Turner et al., 2017; Veenvliet et al., 2020; Vianello and Lutolf, 2020 preprint). Although the reason for this is unclear, the variation may be rooted in ESC genetic backgrounds and/or pluripotency conditions, both linked to the propensity to form the different germ layers in gastruloids (Veenvliet and Herrmann, 2021). We therefore envision that hypoxia could be harnessed to induce gut primordia more robustly in gastruloids and other models of embryo development.

Overall, our gastruloid data point to the importance of using physiologically relevant oxygen concentrations in models of embryo development. This is further corroborated by recent studies demonstrating that hypoxic culture conditions increase both the efficiency of the formation of blastocyst-like structures from human and mouse pluripotent stem cells, and *ex utero* culture of embryos from post-implantation to early organogenesis stages (Aguilera-Castrejon et al., 2021; Sozen et al., 2019, 2021). Thus, in addition to other modulators of the microenvironment, such as medium composition and matrix, we suggest that oxygen tension should be taken into consideration when modeling developmental processes in a dish, or culturing mammalian embryos *ex utero*.

### Limitations of the study

*Hif1a* knockout embryos show defects in somites and the neural tube/fold, which is in agreement with our results of the beneficial effects of hypoxia on enhancing the somite and notochord signatures (Kozak et al., 1997). Although *Hif1a* knockout embryos gastrulate, form the body axis and survive until midgestation, their defects may arise at a much earlier time point, even at gastrulation. Analysis of cellular diversity and identity at gastrulation stages (E6.5-E9.5) in wild-type versus *Hif1a* knockout embryos at the single-cell level will be required to determine the requirement for HIF1α in these processes.

Although spontaneous elongation of gastruloids also occurs in HN −Chi, it is more efficient in the NH −Chi condition. This may be due to higher levels of WNT induction at NH −Chi structures at 72 h and, therefore, timing of peak WNT activity as well as its interplay with SOX2 (Blassberg et al., 2022). The precise amounts of these two regulators are likely dependent on culture conditions (ESC versus differentiation media) and shape the cellular profile in NH −Chi structures, e.g. by promoting WNT-driven differentiation upon exit from pluripotency. Adjustment of hypoxia level, duration and window is likely to further optimize the resulting structures with enhanced morphology as well as lineage representation. In line with this, gradual hypoxia has recently been shown to benefit *ex utero* mouse embryogenesis (Aguilera-Castrejon et al., 2021). Although culture conditions for *ex utero* embryogenesis and gastruloid generation are markedly different, parallel investigation of developmental processes in *ex utero* development and *in vitro* differentiation may provide further insights into the distinct effects of hypoxia at different stages of development.

## MATERIALS AND METHODS

### Cell lines and culture conditions

Wild-type E14 mouse ESCs (provided by Sarah Kinkley, Max Planck Institute for Molecular Genetics, Berlin, Germany) were cultured without feeders on gelatin-coated plates (0.1% gelatin, Sigma-Aldrich, G1393). The media was changed every day and cells were passaged every 2 days. At each passage, cells were dissociated using TrypLE (Thermo Fisher Scientific, 12604-021) and re-plated at the appropriate density. Cells were maintained at 37°C in a humidified 5% CO_2_ incubator. TSCs (provided by Magdalena Zernicka-Goetz, California Institute of Technology, Pasadena, CA, USA) were cultured on feeders using TSC culture media (see below). Cells were passaged in a 1:10 to 1:20 ratio every 4-6 days. XEN cells (provided by Magdalena Zernicka-Goetz) were cultured on gelatin-coated plates using XEN cell culture media (see below). Cells were passaged in a 1:20 or 1:40 ratio every 2-3 days. Cells in normoxia were cultured with 20% O_2_ and cells in hypoxia were cultured with 2% O_2_ unless otherwise specified. Cells were regularly tested for *Mycoplasma* contamination.

### Media and supplements

#### ESC culture media

DMEM/high glucose with Glutamax media (Thermo Fisher Scientific, 31966047) supplemented with 15% fetal bovine serum (FBS) (Thermo Fisher Scientific, 2206648RP), 1× non-essential amino acids, 1× Penicillin/streptomycin, 1× β-mercaptoethanol, 1000 U/ml leukemia inhibitory factor (LIF; homemade).

#### TSC culture media

RPMI 1640 (Gibco, 61870010) supplemented with 1× Glutamax, 20% FBS, 1 mM sodium pyruvate (Thermo Fisher Scientific, 11360070), 1.3× β-mercaptoethanol, 1× penicillin/streptomycin, 25 ng/ml FGF4 (R&D Systems, 235-F4-025) and 1 µg/ml heparin (Sigma-Aldrich, H3149).

#### XEN cell culture media

DMEM+GlutaMAX (Gibco, 10566016) supplemented with 15% FBS, 1× non-essential amino acids, 1× penicillin/streptomycin, 1× β-mercaptoethanol, 1 mM sodium pyruvate, 20 mM HEPES (Sigma-Aldrich, H0887). Media was conditioned on mouse embryonic fibroblasts for 3 days and filtered before use. Then, feeder-conditioned media was mixed in a 7:3 ratio with unconditioned XEN media.

### Inhibitor treatments

For activation of HIF1α, cells were cultured in normoxia and treated with 50 or 100 µM IOX2 (Sigma-Aldrich, SML0652) dissolved in DMSO (Sigma-Aldrich, D2650).

For inhibition of oxidative phosphorylation, cells were cultured in normoxia and treated with 1 µM carbonyl cyanide 4-(trifluoromethoxy)phenylhydrazone (FCCP) (Sigma-Aldrich, C2920) dissolved in DMSO.

For WNT pathway inhibition, cells were treated with 10 µM XAV939 (Sigma-Aldrich, X3004) or 1 µM LGK-974 (BIOTREND, 331-21058-1) for gastruloid formation from aggregation day (0 h) until the end time point (120 h).

### Measurement of lactate

To determine whether iOxPhos increases glycolysis rate, we measured the amount of lactate secreted into the cell culture media using the Lactate-GLO kit (Promega, J5021). E14 ESCs were grown in normoxia or hypoxia (2% O_2_) for 48 h. After 48 h, 10,000 cells were seeded per well of a 96-well cell culture plate coated with 0.1% gelatin (Sigma-Aldrich, G1393). At this point, FBS was substituted with dialyzed FBS (Gibco, A33820). Cells were then treated with 1 µM FCCP or DMSO for 24 h in normoxia or hypoxia. Afterwards, the supernatant was collected, diluted 1:15 in PBS and frozen until use. To determine the lactate amount, the supernatant was mixed with Lactate-GLO assay reagent according to the kit instructions in a final volume of 100 µl. A BMG LABTECH Microplate reader (LUMIstar Omega) was used for readout with the following settings: Method-Luminescence, Mode-Endpoint, Optic-Top, Microplate-GREINER 96-F-BOTTOM, Emission filter lens, Gain-3600, Measurement interval time-1.00, No shaking. PBS and ES media were used as negative control and 100 µM lactate was used as positive control. Three technical and two biological replicates were performed. Luminescence readout was normalized to cell number measured at the time of supernatant collection.

### Growth curve

Cells were grown at a density of 0.25, 0.75 and 0.5 million cells per 10 cm dish for ESCs, TSCs and XEN cells, respectively, in normoxia or hypoxia 2% O_2_, and were counted every day.

### Apoptosis assay

Cells were cultured in normoxia and hypoxia 2% O_2_ for 7 days. Then, the CellEvent Caspase-3/7 Green Flow Cytometry Assay Kit (Thermo Fisher Scientific, C10740) was used. Briefly, cells were collected and incubated with 500 nM CellEvent Caspase-3/7 at room temperature (RT) for 1 h. During the last 5 min, 1 µM SYTOX (from the kit) was added. As negative controls, cells were incubated in the absence of both reagents (double-negative control), only with caspase 3/7 or only with SYTOX (single-negative controls). Cells were analyzed on a FACS AriaFusion. Data were analyzed using FlowJo (version 10) and plotted using GraphPad Prism (version 8).

### Cell cycle analysis

ESCs were cultured in normoxia and hypoxia 2% O_2_ for 7 days. To study cell cycle distribution, the Click-iT EdU Alexa Fluor 488 Flow Cytometry Assay Kit (Thermo Fisher Scientific, C10425) was used. Briefly, on day 7, cells were incubated at 37°C for 1 h with 10 µM 5-ethynyl-2′-deoxyuridine. Following the incubation, 1 million cells were collected and dislodged in fixative for at RT for 15 min. Then, cells were washed and mixed with the reaction cocktail (Alexa Fluor 488 azide) and incubated for 30 min. Cells were treated with 1:1000 DNA content stain FxCycle Violet in FACS media (PBS+1% FBS). Cells were analyzed on a FACS AriaFusion. Data were analyzed using FlowJo (version 10) and plotted using GraphPad Prism (version 8).

### RNA extraction and RT-qPCR

Cells were cultured at matching density and similar confluency to prevent contribution of cell density to the phenotype. Matching normoxia samples were collected for time-course hypoxia analysis. Total RNA was extracted using the QIAGEN RNeasy kit (QIAGEN, 74004), following the manufacturer's instructions and 5 µg of RNA was used as input to generate complementary DNA (cDNA) with the High-Capacity cDNA Reverse Transcription Kit (Thermo Fisher Scientific, 4368814) using random primers. As a control, a reaction without reverse transcriptase enzyme (−RT control) was performed. cDNA was diluted 1:5 and was used to perform real-time quantitative PCR (qPCR) using primers designed to amplify specific target genes (Table S11) using KAPA SYBR FAST qPCR Master Mix (2X) ABI Prism (Thermo Fisher Scientific, KK4617) on the QuantStudio 7 Flex Real-Time PCR System (Applied Biosystems) thermal cycler. As a control, a reaction without cDNA was performed. In RT-qPCR experiments, data represent log_2_FC over normoxia normalized to *Eif1a* and standard deviation for two biological replicates, and a two-tailed, paired Student's *t*-test was applied, unless otherwise indicated.

### Western blotting

#### Whole-cell extracts

Cell pellets were resuspended in RIPA buffer (Thermo Fisher Scientific, 89900) containing 1× protease inhibitor cocktail (Thermo Fisher Scientific, 78425), incubated at 4°C for 30 min, followed by centrifugation at maximum speed (21,000 ***g***) at 4°C for 20 min. Afterward, the supernatant was collected and the protein concentration was determined using Pierce™ BCA™ Protein-Assay (Thermo Fisher Scientific, 23225); 10-20 µg of protein was used for subsequent steps.

#### Subcellular fragmentation of cytoplasm and nucleus

Cell were washed with buffer A [10 mM HEPES, pH 7.9 (Gibco, 15630-080), 5 mM MgCl_2_ (Sigma-Aldrich, M8266), 0.25 M sucrose (Sigma-Aldrich, S7903), 0.1% Igepal 630 (Merck, 56741), 1× protease inhibitor cocktail, 1 mM PMSF, 1 mM NaVO_3_] and incubated on ice for 10 min. Afterwards, samples were passed through 18G needles and centrifuged again for 10 min at 21,000 ***g*** at 4°C. The pellet, corresponding to the nuclear fraction, was resuspended in cold buffer B [10 mM HEPES, pH 7.9, 1 mM MgCl_2_, 0.1 mM EDTA (Jena Bioscience, BU-105), 25% glycerol (Sigma-Aldrich, G5516), 0.5 M NaCl (Invitrogen, AM9760G) and 0.5% Triton X-100] and incubated on ice for 30 min. Samples were passed through 18G needles and sonicated using a Bioruptor with the settings 30 s on 30 s off for 5 min. Subcellular fractions were quantified using Pierce™ BCA™ Protein-Assay; 10-20 µg protein was used for subsequent steps.

#### SDS-PAGE

Samples were mixed with 4× ROTI loading buffer (Carl Roth, K929.2), heated at 98°C for 5 min, and loaded on 4-15% Mini-PROTEAN^®^TGX™ precast protein gels (Bio-Rad, 4561083). Proteins were separated by electrophoresis at 70 V for 15 min followed by 100 V for approximately 1 h using 10× Tris/Glycine/SDS running buffer (Bio-Rad, 1610772).

#### Blotting and detection

Proteins were transferred to a PVDF membrane (Thermo Fisher Scientific, IB24001) using the iBlot 2 dry blotting system (Thermo Fisher Scientific, IB21001) and run at 20 V for 7 min. After blotting, membranes were blocked with 5% milk in TBS-T buffer (Thermo Fisher Scientific, 28360) for 1 h at RT. For detection of the protein of interest, membranes were incubated at 4°C overnight with primary antibody (Table S11) in 5% milk in TBS-T buffer, followed by secondary antibody at RT for 1 h. For detection, membranes were incubated with ECL Western Blotting Substrate (Thermo Fisher Scientific, 32106) for 1 min prior to imaging with the ChemiDoc system (Bio-Rad).

### Immunofluorescence

Immunofluorescence was either performed on colonies grown on glass-bottom chamber slides (Falcon 8-well culture slides, Corning, 354108) or cells/colonies seeded on chamber slides on the day of fixation. Cells were washed with 1× PBS before fixation using 4% paraformaldehyde (PFA) (Sigma-Aldrich, P6148) for 10 min at RT. After fixation, cells were washed with 1× PBS and permeabilized using 0.4% Triton X-100 in PBS supplemented with 0.1% Tween 20 (PBS-T; Thermo Fisher Scientific) for 15 min at RT. After permeabilization, samples were washed in PBS-T and blocked in PBS-T+2% bovine serum albumin (BSA) (NEB, B9000)+5% donkey/goat serum (Jackson ImmunoResearch/Dianova, 017-000-121) for 1 h at RT. Cells were incubated with primary antibodies diluted 1:200 to 1:1000 in blocking buffer overnight at 4°C. Samples were washed three times for 10 min each wash at RT in PBS-T+2% BSA and subsequently incubated with secondary antibodies diluted 1:1000 in blocking buffer for 1 h at RT. Samples were washed three times for 10 min each wash at RT in PBS-T+2% BSA. Cells were mounted in Vectashield (Vector Laboratories, H1200) with DAPI (BIOZOL, VEC-H-2000-10). Imaging was performed with an LSM800 confocal laser scanning microscope (ZEISS). Fluorescence intensities were quantified using CellProfiler (https://cellprofiler.org/) (Carpenter et al., 2006). Intensities were normalized to the nuclear area and plotted in R using ggplot2 (Wickham, 2016). See Table S11 for a full list of antibodies used.

### T reporter activity by flow cytometry

A cell line expressing T:H2B-mCherry was used (Veenvliet et al., 2020). Cells were grown on mouse embryonic fibroblasts in ES media containing serum and LIF for 7 days in normoxia or hypoxia with regular splitting and media changes. After 7 days, cells were dissociated, washed, resuspended in PBS+1% BSA, passed through a cell strainer and collected in FACS tubes. T reporter activity was determined by analyzing cells on a FACS ARIA machine. Data were acquired using BD FACSDiva software, analyzed in FlowJo and plotted in GraphPad Prism.

### Bulk RNA-seq

#### Sample collection

Cells were cultured at matching density and similar confluency to prevent contribution of cell density to the phenotype. Total RNA was extracted using the QIAGEN RNeasy kit and quantified using Qubit 3.0. Two biological replicates were collected per condition.

#### Library preparation and sequencing

Library preparation was performed using a KAPA RNA HyperPrep Kit (Kapa Biosystems, KR1350) (input amount 500 ng RNA, adapter concentration 1.5 µM and PCR cycle number=10), and samples were sequenced using an Illumina HiSeq4000, in 75-bp, paired-end format.

#### Mapping and analysis

Raw reads were subjected to adapter and quality trimming with cutadapt (version 2.4; parameters: --nextseq-trim 20 --overlap 5 --minimum-length 25 --adapter AGATCGGAAGAGC -A AGATCGGAAGAGC), followed by poly-A trimming with cutadapt (parameters: --overlap 20 --minimum-length 25 --adapter ‘A[100]’ --adapter ‘T[100]’). Reads were aligned to the mouse reference genome (mm10) using STAR (version 2.7.5a; parameters: --runMode alignReads --chimSegmentMin 20 --outSAMstrandField intronMotif --quantMode GeneCounts) and transcripts were assembled using StringTie (version 2.0.6; parameters: -e) with GENCODE annotation (VM19). A table of read counts was generated using the featureCounts function of the Rsubread package (version 1.32.4). To filter lowly expressed genes, counts per million (CPM) was calculated using the edgeR package (version 3.26.8). Genes with >0.4 CPM in at least two out of six samples were kept. Additionally, only coding protein genes were used for further analysis. Transcripts per kilobase million (TPM) was used to generate normalized expression values. DE genes were determined using the edgeR package, applying multiple-testing adjusted *P*-value (FDR)≤0.1 significance threshold and absolute FC>1.5. Complete lists of DE genes are available in Table S1.

Principal component analysis (PCA) was performed with the built-in R functions prcomp and hierarchical clustering was completed with the dendextend library (version 1.14.0) using the dist function to compute the distance between sample and hclust. PCA and volcano plots were generated using ggplot2 (version 3.3.0). Heatmaps were generated using GraphPad Prism (version 8).

#### Selection of germ layers and lineage markers

Marker genes from different cell states were selected following the single-cell transcriptional reference profile of early post-implantation development from Grosswendt et al. (2020). Early ecto-, endo- and mesoderm germ layers represent marker genes that were reliably assigned to cell states within these three lineage cell fates from E6.5 to E7.5.

For later cell states, lineage-specific marker genes from node and notochord (E7.0-E8.0, mesoderm lineage), neuromesodermal (E7.5-E8.0, mesoderm lineage), paraxial and posterior mesoderm (E7.5-E8.5, mesoderm lineage), primitive gut (E7.5-E8.5, endoderm lineage), and midbrain and spinal cord (E7.5-E8.5, ectoderm lineage) were selected.

#### Gene ontology-biological process (GO-BP) analysis

GO-BP analyses were performed using DAVID web tools (Dennis et al., 2003)*.* Selected terms were plotted in dot plot format using ggplot2. Complete lists of GO terms are available in Table S2.

#### Analysis of T target genes

Chromatin immunoprecipitation with sequencing (ChIP-seq) and RNA-seq data for T were retrieved from Lolas et al. (2014). Briefly, T-activated (FC>2) or T-repressed (FC<-2) genes in *in vitro* primitive streak differentiated cells on day 4 versus ESCs were selected. Expression levels of these T target genes in hypoxia day 2, hypoxia day 7 and normoxia were plotted in heatmap format using the pheatmap package (version 1.0.12) showing row *z*-score-normalized expression values. Annotation bars were added to show which of these genes are DE in hypoxia day 2 and/or day 7 compared with normoxia.

### Global proteomics

#### Sample preparation

Proteomics sample preparation was carried out according to a published protocol with minor modifications (Kulak et al., 2014). In brief, 5 million cells in biological duplicates of 2 days and 7 days hypoxia treatment and normoxia controls were lysed under denaturing conditions in 500 µl of a buffer containing 3 M guanidinium chloride (GdmCl), 10 mM tris(2-carboxyethyl)phosphine, 40 mM chloroacetamide and 100 mM Tris-HCl, pH 8.5. Lysates were denatured at 95°C for 10 min shaking at 1000 rpm in a thermal shaker and sonicated in a water bath for 10 min, then 100 µl lysate was diluted with a dilution buffer containing 10% acetonitrile and 25 mM Tris-HCl, pH 8.0, to reach a 1 M GdmCl concentration. Then, proteins were digested with LysC (Roche; enzyme-to-protein ratio 1:50, MS-grade) with shaking at 700 rpm at 37°C for 2 h. The digestion mixture was diluted again with the same dilution buffer to reach 0.5 M GdmCl, followed by tryptic digestion (Roche; enzyme-to-protein ratio 1:50, MS-grade) and incubation at 37°C overnight in a thermal shaker at 700 rpm. Peptide desalting was performed according to the manufacturer's instructions (Pierce C18 Tips, Thermo Scientific). Desalted peptides were reconstituted in 0.1% formic acid in water and further separated into four fractions by strong cation exchange chromatography (SCX, 3M Purification). Eluates were first dried in a SpeedVac, then dissolved in 5% acetonitrile and 2% formic acid in water, briefly vortexed, and sonicated in a water bath for 30 s prior to injection to nano-liquid chromatography with tandem mass spectrometry (LC-MS/MS).

#### Run parameters

LC-MS/MS was carried out by nanoflow reverse-phase liquid chromatography (Dionex Ultimate 3000, Thermo Scientific) coupled online to a Q-Exactive HF Orbitrap mass spectrometer (Thermo Scientific), as reported previously (Ni et al., 2019). Briefly, the LC separation was performed using a PicoFrit analytical column (75 μm ID×50 cm long, 15 µm Tip ID; New Objectives) in-house packed with 3-µm C18 resin (Reprosil-AQ Pur, Dr. Maisch).

#### Peptide analysis

Raw MS data were processed with MaxQuant software (v1.6.10.43) and searched against the mouse proteome database UniProtKB with 55,153 entries, released in August 2019. The MaxQuant processed output files can be found in Table S3, showing peptide and protein identification, accession numbers, percentage sequence coverage of the protein, q-values, and label-free quantification (LFQ) intensities. The MS data have been deposited to the ProteomeXchange Consortium (http://proteomecentral.proteomexchange.org) via the PRIDE partner repository (Martens et al., 2005) with the dataset identifier PXD026641.

#### DE analysis

The DEP package (version 1.6.1) was used. First, duplicate proteins, contaminants, and proteins that were not found in at least two out of the total number of samples (*n*=6) were filtered. A total number of 4260 proteins were identified. LFQ values were normalized using the background correction variance stabilizing transformation. Missing values were imputed using a left-censored imputation method as the proteins with missing values were biased to low expression values. DE analysis was run with test_diff function from the DEP package, which uses limma (Ritchie et al., 2015). DE proteins were defined applying multiple-testing adjusted *P*<0.05 (Benjamini–Hochberg) significance threshold and FC with an absolute value of >1.5. Complete lists of DE proteins are available in Table S4.

Scatter plots show mean LFQ values in hypoxia versus normoxia and were generated using ggplot2. GO-BP analysis was performed using DAVID web tools (Dennis et al., 2003).

### ChIP-seq

Normoxia, hypoxia (2 days) and hypoxia (6 days) samples were used. Chromatin extracts were prepared as described by Brookes et al. (2012). Briefly, cells were fixed with 1% formaldehyde (Thermo Fisher Scientific, 28906) in PBS at RT for 10 min. Then, 0.125 M glycine (Sigma-Aldrich, 50046) was added to quench the formaldehyde at RT for 5 min. Cells were washed twice with ice-cold PBS. To lyse, fixed cells were treated with swelling buffer [25 mM HEPES, pH 7.9, 1.5 mM MgCl_2_, 10 mM KCl (Invitrogen, AM9640G), 0.1% Igepal 630, 1× protease inhibitor cocktail, 1 mM PMSF, 2 mM NaVO_3_, 5 mM NaF] at 4°C for 10 min. Cells were scraped on ice and passed through 18G needles before centrifugation at 3000 ***g***, at 4°C for 5 min. The cell pellet, corresponding to nuclei, was carefully resuspended in sonication buffer [50 mM HEPES, pH 7.9, 140 mM NaCl, 1 mM EDTA, 1% Triton X-100, 0.1% sodium deoxycholate (Thermo Fisher Scientific, 89904), 0.1% SDS (Invitrogen, AM9822), 1 mM PMSF, 2 mM NaVO_3_, 5 mM NaF] and incubated on ice for 10 min before sonication. Chromatin was sheared to an average size of 200-300 bp with an E220 Evolution Covaris sonicator for six cycles, 1 min each. Shearing efficiency was checked by agarose gel.

Chromatin (10 µg) was incubated with 5 µl of HIF1α antibody (concentration not provided) (Cell Signaling Technology, 36169, lot 2), 20 µl of Protein A/G dynabeads (Thermo Fisher Scientific, 10002D/10004D) in sonication buffer overnight. Beads were washed twice with sonication buffer, followed by a wash with high-salt buffer (sonication buffer with 500 mM NaCl instead) and TE buffer [10 mM Tris-HCl, pH 8.5 (Teknova, T5085), 1 mM EDTA, 1% SDS]. Then, the sample was resuspended in elution buffer [50 mM Tris-HCl, pH 7.5 (Sigma-Aldrich, T2319), 1 mM EDTA, 1% SDS]. Samples were treated with RNase A (Thermo Fisher Scientific, EN0531) at 37°C for 30 min, followed by Proteinase K (NEB, P8107S) at 65°C overnight. Genomic DNA was purified using MinElute PCR purification kit (QIAGEN, 28004) and quantified using Qubit 3.0. Two biological replicates were collected per condition.

#### Library preparation and sequencing

Libraries were prepared using the KAPA Hyper Prep Kit (Kapa Biosystems, KR0961) (input amount 10 ng DNA, adapter concentration 1.5 µM, and size selection of 200-700-bp after PCR with cycle number=15). Samples were sequenced using NovaSeq 6000, in 100-bp, paired-end format.

#### Mapping and analysis

Raw reads of treatment and input samples were subjected to adapter and quality trimming with cutadapt (Martin, 2011) (version 2.4; parameters: --nextseq-trim 20 --overlap 5 --minimum-length 25 --adapter AGATCGGAAGAGC -A AGATCGGAAGAGC). Reads were aligned to the mouse genome (mm10) using BWA with the ‘mem’ command (version 0.7.17, default parameters). A sorted BAM file was obtained and indexed using samtools with the ‘sort’ and ‘index’ commands (version 1.10). Duplicate reads were identified and removed using GATK (version 4.1.4.1) with the ‘MarkDuplicates’ command and default parameters. Peaks were called with reads aligning to the mouse genome only using MACS2 ‘callpeak’ (version 2.1.2; parameters --bdg --SPMR) using the input samples as control samples. For downstream analyses, after validation of reproducibility, replicates were pooled using samtools ‘merge’. Genome-wide coverage tracks (signal files) for merged replicates normalized by library size were computed using samtools bamCoverage (version 3.4.3) (parameters: --normalizeUsing CPM --extendReads) When required, bedgraph files were also generated using bigWigToBedGraph from Kent utils tools (Kent et al., 2010)*.* For identification of consistent HIF1α peaks, only those that were identified in both replicates were used for downstream analyses. Peaks were annotated using ChIPseeker package (version 1.20.0) using default parameters (TSS region ±3 kb) and subdivided into peaks at promoter or distal regions. A complete list of HIF1α peaks is available in Table S6.

Density plots were generated using computeMatrix (reference-point --referencePoint center) and plotHeatmap functions from deepTools. Enrichment of different features was at ±8 kb of centered HIF1α peaks at promoters or distal regions. H3K27ac, p300, H3K4me3 and H3K4me1 ChIP-seq data generated on E14 ESCs were retrieved from Cruz-Molina et al. (2017)*.*

Genome views of selected loci were generated using Spark (version 2.6.2). β-Catenin ChIP-seq data generated on V6.5 ESCs were retrieved from Zhang et al. (2013) and analyzed following our ChIP-seq workflow.

Overlap of HIF1α-, β-catenin- and ESC enhancer peaks was generated using intersect function (-wa -wb) from bedtools (version 2.29.2). Venn diagrams were generated manually. A complete list of HIF1α/β-catenin common targets at promoters and enhancers is available in Table S8.

### Gastruloid formation

Male F1G4 mouse ESCs (George et al., 2007) were cultured on 6 cm plates (Corning, 430166) gelatinized with 0.1% gelatin (Sigma-Aldrich, G1393) and coated with mitotically inactive primary mouse embryonic fibroblasts (3-4×10^4^ cells/cm²) with standard ES medium containing 15% fetal calf serum and 1000 U/ml LIF (Chemicon, ESG1107) at 37°C and 5% CO_2_. ESCs were split every second day with a 1:10 dilution. For splitting, media was aspirated and cells were washed once with PBS and trypsinized [Trypsin-EDTA (0.05%) (Gibco 25300054)] for 5-10 min at 37°C. Trypsin was neutralized by 3 ml ES media and cells centrifuged for 5 min at 1000 ***g***, after which the pellet was resuspended in ES media. ESCs were cultured in normoxia or hypoxia for 7 days. Gastruloids were then generated as described previously (Veenvliet et al., 2020) with some minor modifications. Briefly, ESCs were first depleted of feeders, then washed once in 5 ml pre-heated (37°C) PBS containing MgCl_2_ and CaCl_2_ (Sigma-Aldrich, D8662) and once in 5 ml NDiff 227 medium (Takara Bio, Y40002) pre-conditioned in normoxia or hypoxia. ESCs were then pelleted by centrifugation for 5 min at 1000 ***g*** and resuspended in 250 µl NDiff 227. Then, 10 µl of the cell suspension was mixed with 10 µl of Trypan Blue (Bio-Rad, 1450021) for automated cell counting with a Luna Automated Cell Counter. Four-hundred live cells were plated in a volume of 35 µl NDiff 227 into each well of a 96-well, round-bottom, low-attachment plate (Costar 7007 ultra-low-attachment 96-well plates). Cells were then allowed to aggregate for 48 h under normoxic or hypoxic conditions. After 48 h, the aggregates were treated with 3 µM Chi (CHIR99021, Merck Millipore) in 150 µl NDiff 227 for 24 h to induce robust gastruloid formation. For −Chi aggregates, 150 µl NDiff 227 without Chi was added. Between 72 and 120 h, medium was refreshed every 24 h by removing 150 µl of the old media and adding the same volume of new, pre-conditioned NDiff 227. Hypoxic gastruloids were formed at 2% O_2_.

### Whole-mount immunofluorescence of gastruloids

Gastruloids were picked using a p200 pipette with the tip cut off at the 50 µl mark. Gastruloids were washed twice with PBS+MgCl_2_ and CaCl_2_+0.5% BSA (Sigma-Aldrich, A8412), once with PBS, and then fixed in 4% PFA for 75 min in 8-well, glass-bottom plates (ibidi, 80827) at 4°C on a rocking platform. Subsequently, gastruloids were washed twice in PBS for 5 min, permeabilized by incubating for three 10 min washes in 0.5% Triton X-100/PBS (PBST), and blocked in 10% fetal calf serum/PBS-T (blocking solution) overnight at 4°C. Primary antibody incubation was performed in blocking solution for 48-72 h at 4°C, after which gastruloids were washed three times with blocking solution and three times with PBS-T. The following primary antibodies were used: rabbit anti-T (Cell Signaling Technology, D2Z3J; 1:500), goat anti-SOX2 (R&D Systems, AF2018; 1:500), goat anti-FOXA2 (Santa Cruz Biotechnology, sc-6554; 1:500). After the last washing step, gastruloids were incubated in blocking solution overnight at 4°C. The next day, secondary antibodies diluted in blocking solution were added, and gastruloids were incubated for 24 h at 4°C. The following secondary antibodies were used, all at a dilution of 1:500: donkey anti-rabbit Alexa Fluor 647 (Thermo Fisher Scientific, A31573), donkey anti-goat Alexa Fluor 546 (Thermo Fisher Scientific, A11056). Afterwards, gastruloids were washed three times with blocking solution and three times with PBST. The last PBST washing step after secondary antibody incubation included DAPI (0.02%; Roche Diagnostics, 10236276001). DAPI was incubated for 5 h or overnight and washed off once with PBS.

### Clearing and imaging of gastruloids for confocal microscopy

Prior to imaging, gastruloids were embedded in agarose and cleared with RIMS [Refractive Index Matching Solution; 133% w/v Histodenz (Sigma-Aldrich, D2158) in 0.02 M PB (see below)]. To this end, 10% low melting point (LMP), analytical grade (Promega, V2111) agarose was prepared in PBS, incubated at 80°C for 15 min, and cooled down to 37°C for 3 min in a thermomixer. Samples were washed twice with PBS for 10 min, post-fixed in 4% PFA for 20 min, and washed three times with 0.1 M phosphate buffer (PB; 0.025 M NaH_2_PO_4_, 0.075 M Na_2_HPO_4_, pH 7.4). The pipette was set to 20 µl and the gastruloids were stabilized on the ibidi plate with a drop of LMP agarose for 5 min until the agarose was dry. Clearing was performed by incubation in 200 µl RIMS on a rocking platform at 4°C for one to several days. Cleared gastruloids were imaged with a ZEISS LSM 880 Airyscan in confocal, Airyscan or Fastairy mode, using a Plan-Apochromat 20×/NA=0.8 objective, lateral pixel size of 0.1.2-1.2 µm and a typical *z*-spacing ranging from 1.9 to 3.3 μm and appropriate laser/filters for DAPI, Alexa Fluor 546 and Alexa Fluor 633 or combinations thereof. Brightfield images of non-cleared gastruloids were acquired with a ZEISS Celldiscoverer 7, with a Plan-Apochromat 5×/NA=0.3 objective and a 1× post-magnification, lateral pixel size of 0.9 µm and a typical *z*-spacing of 10 μm, running under ZEN Blue v3.1.

### Light-sheet microscopy

The gastruloids were imaged using a ZEISS Lightsheet LS Z1 microscope with appropriate filters for mCherry, Alexa 488 and DAPI. Prior to imaging, the gastruloids were embedded in 1.5% LMP agarose in a glass capillary and kept in the fridge for 5 min until the agarose was solidified. Subsequently, the capillary containing the samples in agarose columns were placed into the RIMS-filled sample chamber of the ZEISS Lightsheet Z1. The agarose column was slightly pushed out of the capillary into the RIMS solution and left overnight to clear the gastruloids. Post-processing of the images was performed using ZEN Blue/Black software (ZEISS).

### Post-acquisition image processing and analysis

Images captured in airy mode were processed using ZEN Black 2.3 software on a dedicated workstation. Confocal and wide-field image sets were analyzed downstream and further processed using ZEN Blue (version 3.2). Maximum intensity projections (MIPs) for morphometric analysis of gastruloids were generated using customized macros in the open application development module in ZEN Blue v3.2. Morphometric analysis was performed by either variance-based thresholding (brightfield images) or Otsu intensity thresholding (confocal MIPs) after faint Gaussian smoothing; close-by objects were segmented by standard shedding.

Single-cell image analysis of confocal datasets was also performed with customized analysis pipelines written in the image analysis module in ZEN Blue. Briefly, individual cells were identified by nuclear counter staining after Gaussian smoothing and background subtraction, adjusted to actual resolution of individual datasets, and close-by objects were segmented by water shedding. All objects/nuclei were filtered after identification by area of 100-1000 µm^2^ and circularity of 0.6-1 [√(4×area/π×FeretMax²)]. Within the resulting regions of interest (cells), fluorescence signal of the counterstaining was quantified. In total, >10 million single objects were analyzed, plotted and further quantified using customized R scripts.

### Quantification of T expression across the A-P axis

T expression along the anterior-posterior axis was performed in ZEN 3.3 lite. In detail, MIPs previously generated by ZEN Blue v3.2. were loaded into ZEN 3.3 lite. Brightness/contrast were automatically adjusted and a line (stroke thickness: 1) was manually drawn from the anterior to the posterior end of the structure along the midline, and the fluorescence intensity was measured using the ‘Profile’ function on the software. The distance was measured in nm.

To obtained binned intensities, first relative positions were calculated by dividing the absolute position by the total length of the structure, resulting in standardized relative positions on a 0-1 scale. Average intensities were then calculated for 0.01 sized bins, resulting in average intensity for 100 bins over the A-P axis. Plotting and k-means clustering was performed using PlotTwist (Goedhart, 2020).

### Live imaging

Gastruloids generated from T::H2B-mCherry, Sox2::H2B-Venus double-reporter mouse ESCs were imaged for 15 h using Olympus IXplore SpinSR microscope at 37°C, 5% CO_2_ and 2% O_2_. Imaging was performed by multi-position acquisition of gastruloids with a total stack size of 280 µm using an Olympus UPlanSApo 10×/0.40 objective. mCherry signal was acquired with the 561 nm laser (15% power) and a step size of 10 µm. Brightfield images were acquired with a step size of 70 µm. The imaging interval was set at 35 min.

### Imaging analysis

For morphometric analysis, brightfield images were used. For images acquired using the Olympus IXPlore SpinSR, *z*-stacks were first patched into a single focused image using the Gaussian-based stack focuser plugin. Images were then auto-thresholded using the Otsu algorithm and binary processed to fill holes. Manual thresholding was carried out in samples where auto-thresholding failed. Gastruloids were segmented, and masks with registered regions of interests were created.

For generation of the movie, the projected mCherry images' intensity range of display was set to a minimum of 0 and a maximum of 400, before being merged with the corresponding focused brightfield image. All bioimage analyses were executed using Fiji v1.53 s.

### Multiplexed scRNA-seq

Single-cell transcriptome profiling of gastruloids using Multi-seq was performed by combining protocols described previously (Bolondi et al., 2021; McGinnis et al., 2019; Veenvliet et al., 2020) with some modifications.

Gastruloids from NN +CHI, NH +CHI and NH −Chi conditions were generated as described above. From each condition, 28 elongated structures were pooled. Gastruloids were picked with a p200 pipette with the pipette tip cut-off at the 50 µl mark, and pooled in a 1.5 ml tube filled with ice-cold PBS. The three pools were washed twice with ice-cold PBS. Next, structures were dissociated in 50 µl TrypLE Express (Gibco) for 20 min at 37°C, with pipetting after 10 min. In the meantime, a 10× lipid-modified oligonucleotides (LMOs)–barcode (BC) oligo solution was prepared (1:1; 2 µM each) in PBS for each of the three samples on ice. From here on, every step was performed on ice. After cell dissociation, LMOs–BC solutions were added to each sample separately (final concentration 200 nM), and samples were incubated for 5 min on ice. In the meantime, a 10× co-anchor solution was prepared (2 µM) in PBS. Following the 5 min incubation, the co-anchor solution was added to each sample (final concentration 200 nM), and samples were further incubated for 5 min on ice. The reaction was quenched by adding 1 ml PBS+1% BSA to each tube. Cells were washed twice with 1 ml PBS+1% BSA with centrifugation steps performed for 5 min at 300 ***g*** and 4°C in low DNAbind Eppendorf tubes. Cell pellets were resuspended in 100 µl PBS+0.4% BSA and pooled together in one new tube. The cell suspension was filtered using Scienceware Flowmi Cell Strainers, 40 µm. Cells were then centrifuged for 5 min at 300 ***g*** at 4°C, and resuspended in 45 µl PBS+0.4% BSA and the cell concentration was determined using a hemocytometer. Cells were subjected to scRNA-seq (10x Genomics, Chromium™ Single Cell 3′ v3; one reaction). Single-cell libraries were generated according to the manual, with one modification: Multi-seq additive primer (5′-CTTGGCACCCGAGAATTCC-3′) was added at the cDNA amplification step (for a detailed protocol, see McGinnis et al., 2019). During amplified cDNA cleanup, the Multi-seq BC fraction was isolated and a separate library was performed as described. The cDNA library was sequenced with a minimum of 400 million fragments and Multi-seq BC library was sequenced with a minimum of 50 million fragments.

### scRNA-seq analysis

All analyses and plots were generated using R version 4.1.0 ‘Camp Pontanezen’ and Seurat (version 4.0.5) (Hao et al., 2021).

#### Preprocessing

The Cell Ranger pipeline version 3 (10x Genomics) was used for scRNA-seq data set to de-multiplex the raw base call files, generate fastq files, perform the mapping to the mouse reference genome mm10, filter the alignment and count barcodes and unique molecular identifiers.

To de-multiplex samples within our single Multi-seq scRNA-seq dataset, we used the deMULTIplex R package (version 1.0.2) (McGinnis et al., 2019). In short, sample IDs, ‘GGAGAAGA’ for NN +Chi, ‘CCACAATG’ for NH +Chi and ‘TGAGACCT’ for NH –Chi, were assigned to cells. Cells with no associated sample barcode and cells with more than one barcode (doublets) were discarded for downstream analysis.

#### Quality control

The initial quality control was performed with Seurat. Single-cell data generated were loaded with a minimum requirement of three cells and 200 features (default parameters). Cells with fewer than 200 or more than 2500 unique feature counts and a mitochondrial fraction above 5% were removed from the analysis. Quality control features were checked for each individual condition.

#### Data integration and cluster determination

The filtered Seurat object was divided by condition (NN +Chi, NH +Chi and NH –Chi). Subsequently, the expression data were independently normalized, and variable features within the three datasets were detected, log-normalized and scaled to 10,000 (default settings).

For integration of the three conditions, integration anchors within our three data sets were calculated and a PCA (reduction ‘rpca’, dims=1:30) was run. Finally, these anchors were used to integrate de data. A list of cell cycle markers was used to score for cell cycle stage and to subsequent scale the data with regression out (var.to.regress) of S and G2M phase-related genes (Macosko et al., 2015). For downstream analysis and visualization of the integrated dataset, a PCA followed by a UMAP (dims=1:30, n.neighbors=10) were run. A total number of ten clusters were identified applying standard parameters (FindNeighbors, dims=1:20 and FindClusters, resolution=0.5). These Seurat clusters were further annotated to germ layer origin taking into account cell-type annotation to mouse embryo.

#### Cell-type annotation to mouse embryo

Two independent publicly available mouse reference atlases (E6.5 to E8.5) were used to predict the *in vivo* cell ID of our integrated gastruloid data set as well as for proportion comparisons (Grosswendt et al., 2020; Pijuan-Sala et al., 2019). The reference atlases were filtered to include only the relevant time points for our study (E7, E7.5 and E8.5) and to exclude all extra-embryonic cell states. The mouse reference atlases and our integrated gastruloid data set were normalized (SCTransform, with default parameters). Cell-type classification was performed subsequently by finding anchors to transfer (dims=1:30) and adding these predictions to our integrated gastruloid data set. Prediction scores and percentage of cells assigned to each cluster were used as measurements for the cell-type annotation call (data not shown). Finally, gastruloid cells were matched to their *in vivo* counterparts. All analyses described below were carried out with gastruloid cells assigned to their *in vivo* counterparts according to Grosswendt et al. (2020).

Additionally, we used this information to annotate Seurat clusters based on germ layer origin.

#### Neural cell categorization

All neural cells assigned to future spinal cord and fore/midbrain were extracted from the integrated data set. To classify them, first neural cells were assigned to the Hox-positive module if expressing at least one Hox gene out of a subset of 28 (Duboule and Dollé, 1989). Among Hox-negative cells, a putative brain-like module was defined as cells not expressing any Hox gene, expressing at least five fore/midbrain marker genes out of the top 30 *in vivo* fore/midbrain marker genes, and not expressing the spinal cord marker gene *Fgfbp3* (Grosswendt et al., 2020). Lastly, a spinal cord-like module was defined that corresponded to the remaining fraction of cells that were Hox-negative and expressing fewer than five fore/midbrain marker genes.

#### Dorsal-ventral patterning categorization

All spinal cord assigned cells were extracted from the integrated data set. To study their dorsal-ventral patterning, a list of marker genes belonging to dorsal, midline and ventral identity was used (Sagner and Briscoe, 2019). Th ventral module was defined as cells expressing at least one out of three ventral genes and not expressing midline or dorsal markers. The same rule applied for midline and dorsal module assignment. Around 71.5% of spinal cord assigned cells survived the dorsal-ventral patterning categorization.

#### Pseudo-bulk expression analysis

To calculate average gene expression values per cell state and sample, the ‘AverageExpression’ function in Seurat was used.

### Statistical tests

For all distinct statistical tests performed in this study, *P*-values are given as: *P*>0.05 (not significant, ns), *P<0.05, **P<0.01, ***P<0.001, ****P<0.0001.

## Supplementary Material

10.1242/develop.200679_sup1 Supplementary information
