# Supplementary material for: Hypoxia induces an early primitive streak signature, enhancing spontaneous elongation and lineage representation in gastruloids
Source: Development. 2022 Sep 14;149(20):dev200679. doi: 10.1242/dev.200679 (PMC9578691; doi:10.1242/dev.200679)
Supplement: Supplementary information [file develop-149-200679-s1.pdf]

Figure S1

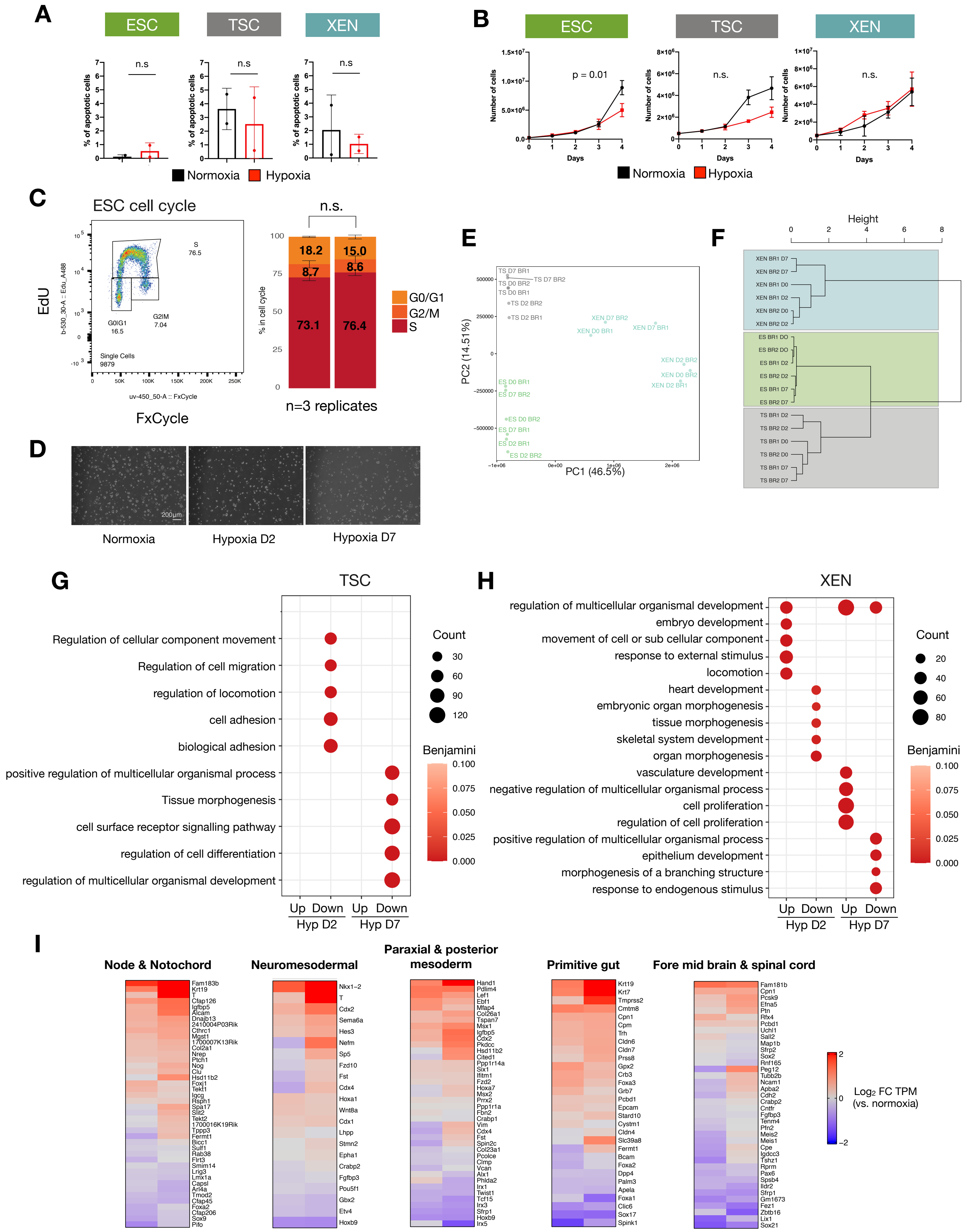

Development • Supplementary information

Fig. S1. Further characterization of the hypoxic response in stem cells.

A) Apoptosis levels of normoxic or hypoxic (d7) ES, TS, and XEN cells, defined as Casp3/7^{+} and Sytox^{-}. See methods for more details. Two biological replicates were performed. Two-tailed paired Student's t-test was applied.

B) Growth curves of ES, TS, and XEN cells grow in normoxia or hypoxia. same number of cells were seeded in both conditions and cells were counted daily. Two biological replicates were performed. The statistical test performed is a two-tailed paired Student's t-test.

C) Analysis of cell cycle phase distribution of normoxic or hypoxic (d7) ESCs. Cells were pulse-labeled with EdU, and EdU vs FxCycle Violet (DNA stain) amounts were measured by flow cytometry. A representative readout (left) and cell cycle distributions (right) are shown. n.s.=non-significant.

D) Bright-field pictures of ESC colonies on the day of collection for bulk RNA-seq.

E, F) Principal component analysis (PCA) (E) and hierarchical clustering (F) based on global transcriptomes.

G, H) GO-BP terms associated with DE genes in TS (G) and XEN (H) cells exposed to acute (d2) or prolonged (d7) hypoxia. Representative significant terms are shown. No significant terms were retrieved for upregulated genes in TS cells on day 2 of hypoxia.

I) Heatmaps showing expression levels of the indicated genes in hypoxic relative to normoxic ESCs. See Methods for details.

Development • Supplementary information

Figure S2

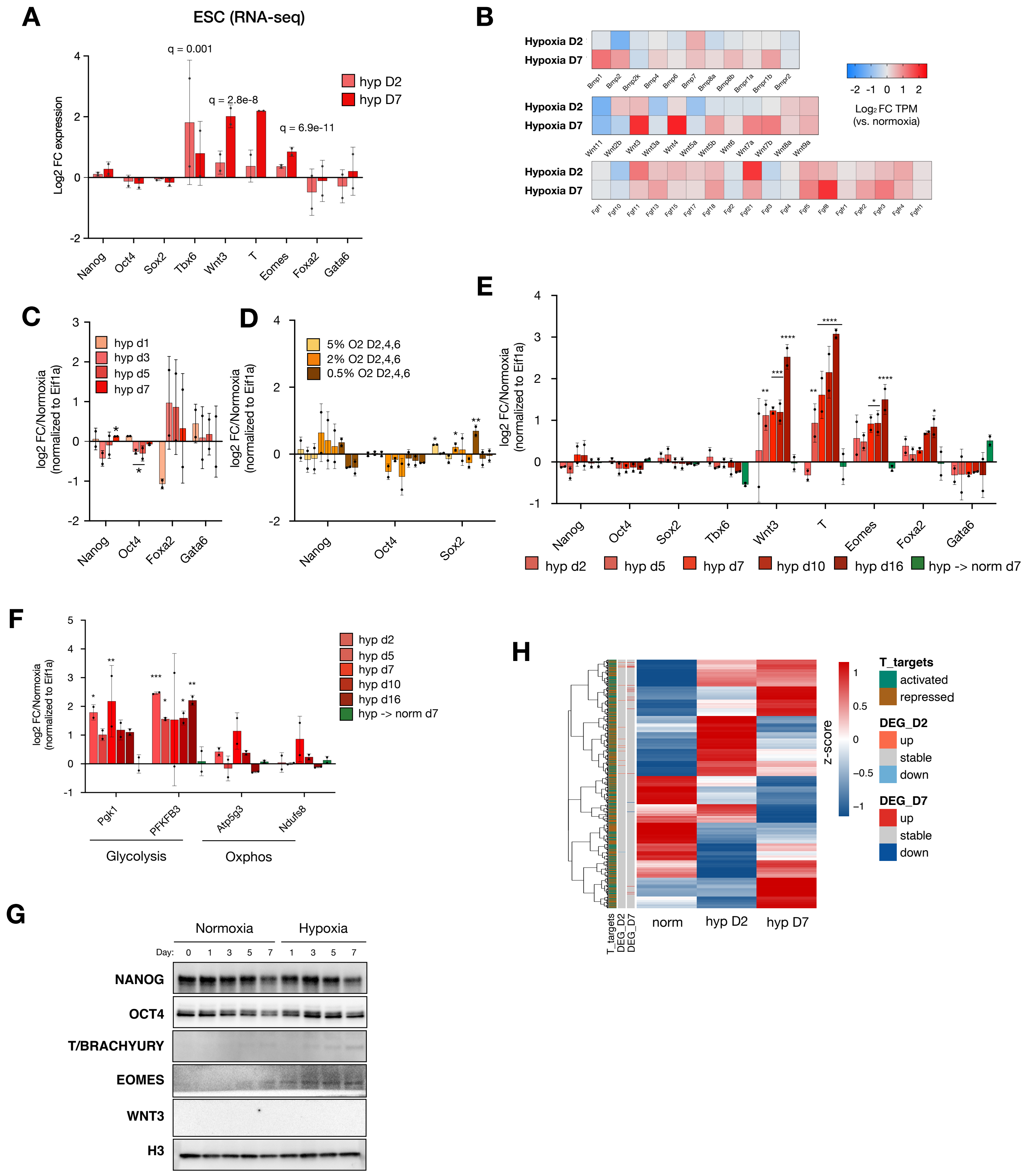

Development • Supplementary information

Fig. S2. Further characterization of pluripotency and differentiation-associated gene activity.

A) Expression levels of shown genes measured by RNA-seq. Data represent log_2FC over normoxic ESCs of TMP expression values and standard deviation.

B) Heatmaps showing expression levels of Bmp, Wnt, and Fgf, genes in hypoxic relative to normoxic ESCs.

C-F) RT-qPCR analysis of shown genes in hypoxic ESCs. Statistical test is two-way ANOVA.

G) Western blot showing expression levels of the indicated genes in normoxic and hypoxic ESCs. H3 was used as a loading control.

H) Heatmap showing expression levels of T target genes in normoxic and hypoxic ESCs. T target genes were identified as described in the Methods. Bar annotations show, 1) T_targets; T -activated (in green) and -repressed (in brown) target genes in *in vitro* primitive streak differentiated cells, 2) DEG in hypoxia day 2 (D2) and day 7 (D7); up- (in red), down- (in blue) regulated and stable (in gray) genes on hypoxia day 2 and/or day 7.

Figure S3

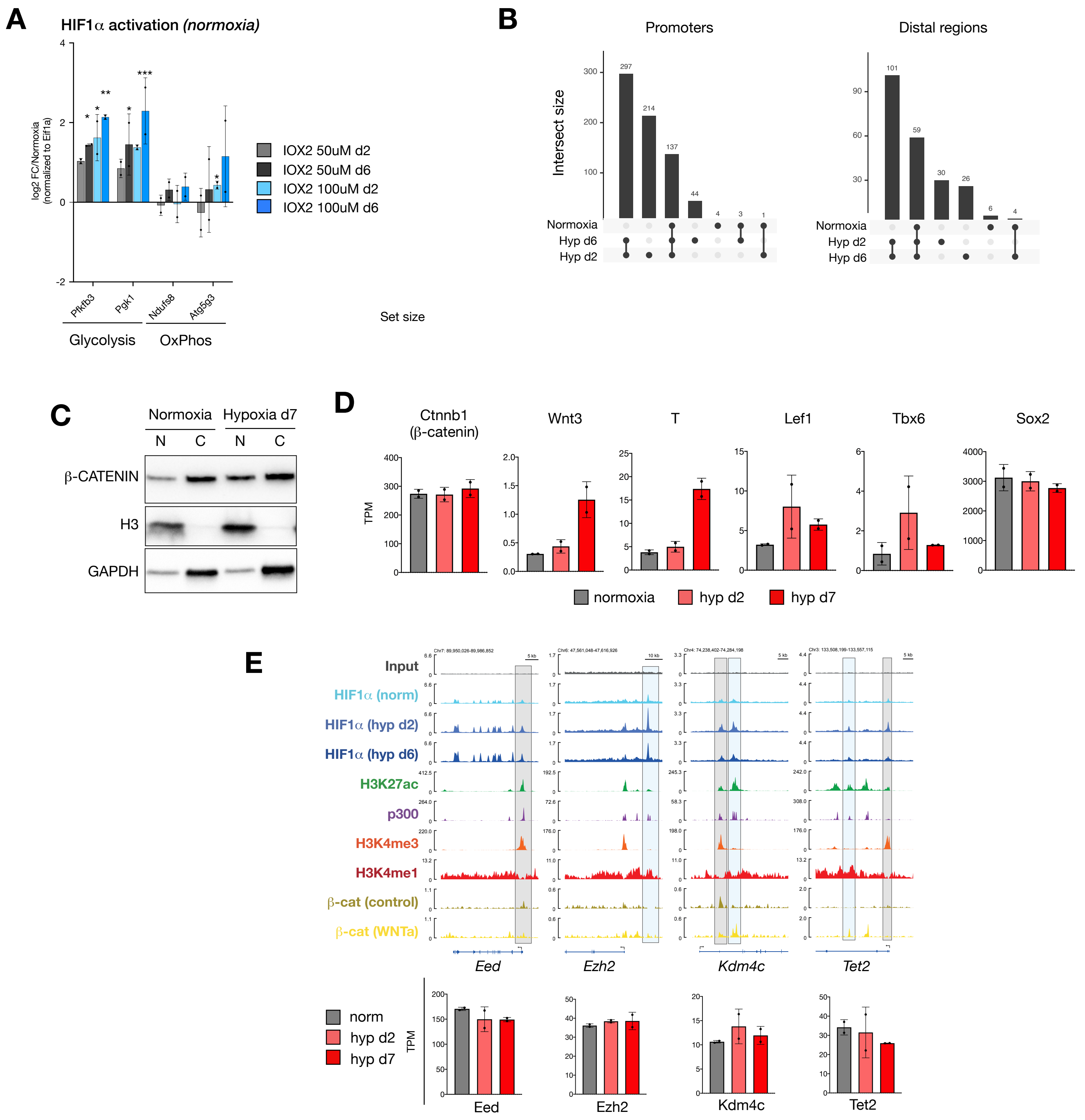

Development • Supplementary information

Fig. S3. Further characterization of Hif1α activity at chromatin.

A) RT-qPCR analysis of shown genes in ESCs treated with the HIF1α activator IOX2 at indicated concentrations in normoxia. Data represent log_2FC over DMSO-treated ESCs. Statistical test is two-way ANOVA.

B) Overlap of HIF1α-bound promoters (left) and distal sites (right) in normoxic and hypoxic ESCs.

C) Protein expression levels and subcellular localization of β-CATENIN in normoxic and hypoxic (d7) ESCs. Histone H3 is used as loading control. H3 and GAPDH were used as loading and fractionation controls. N, nuclear fraction. C, cytoplasmic fraction.

D) TPM expression values of the shown genes in normoxic and hypoxic ESCs as measured by RNA-seq are plotted as bar plots.

E) Genome browser views of HIF1α chromatin occupancy and histone modifications at epigenetic regulators (top). Expression values of the shown genes in normoxic and hypoxic ESCs as measured by RNA-seq are plotted as bar plots (bottom). Gray and blue highlights indicate promoter and ES active enhancers, respectively.

Figure S4

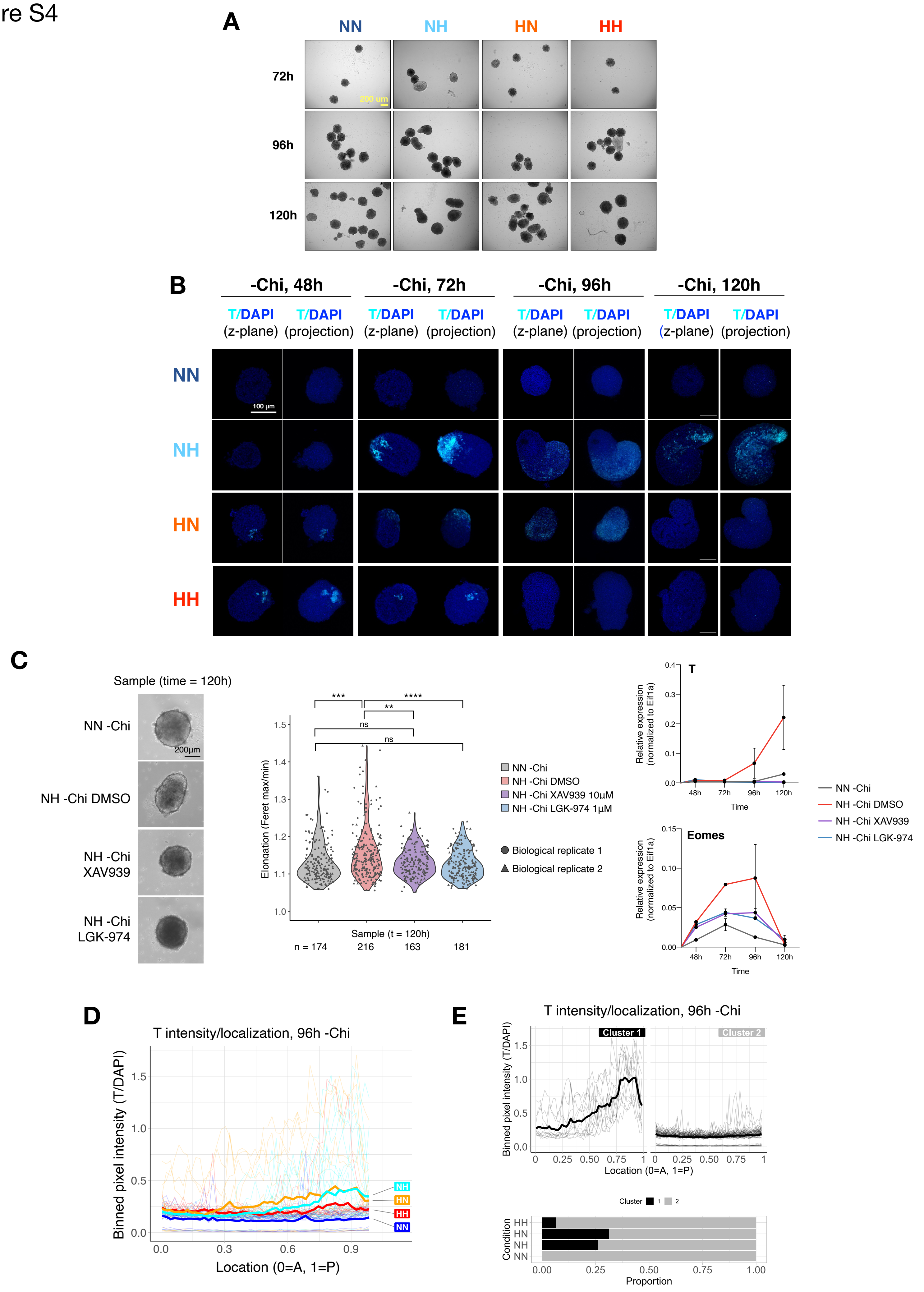
A
NN
NH
HN
HH
72h
96h
120h
200 um
B
-Chi, 48h
-Chi, 72h
-Chi, 96h
-Chi, 120h
T/DAPI (z-plane)
T/DAPI (projection)
NN
NH
HN
HH
100 µm
C
Sample (time = 120h)
NN -Chi
NH -Chi DMSO
NH -Chi XAV939
NH -Chi LGK-974
200µm
Elongation (Feret max/min)
Sample (t = 120h)
n = 174
216
163
181
***
****
**
ns
ns
NN -Chi
NH -Chi DMSO
NH -Chi XAV939 10uM
NH -Chi LGK-974 1µM
Biological replicate 1
Biological replicate 2
T
Eomes
Relative expression (normalized to Eif1a)
Time
48h
72h
96h
120h
NN -Chi
NH -Chi DMSO
NH -Chi XAV939
NH -Chi LGK-974
D
T intensity/localization, 96h -Chi
Binned pixel intensity (T/DAPI)
Location (0=A, 1=P)
NH
HN
HH
NN
E
T intensity/localization, 96h -Chi
Cluster 1
Cluster 2
Binned pixel intensity (T/DAPI)
Location (0=A, 1=P)
Cluster
Condition
Proportion

Fig. S4. Characterization of spontaneously elongating hypoxic gastruloids.

A) Bright-field images of representative gastruloids at the indicated time points.

B) Confocal fluorescent microscopy images of representative -Chi gastruloids at indicated time points of culture.

C) WNT inhibitor treatment of NH- Chi gastruloids. Bright-filed images of representative gastruloids at 120h at the indicated conditions (left), elongation index (middle), and relative expression levels of T and Eomes (right) are shown. Data represent two biological replicates.

D) K-means clustering of the NH and HN structures presented in C) with n=2 clusters.

E) Localization of T signal along the anterior-posterior (A-P) axis of gastruloids in each condition at 96h of culture. T signal normalized to DAPI and binned at 1% length increments along each structure for plotting. Thick lines show mean values and thin lines show data from individual structures.

Development • Supplementary information

Figure S5

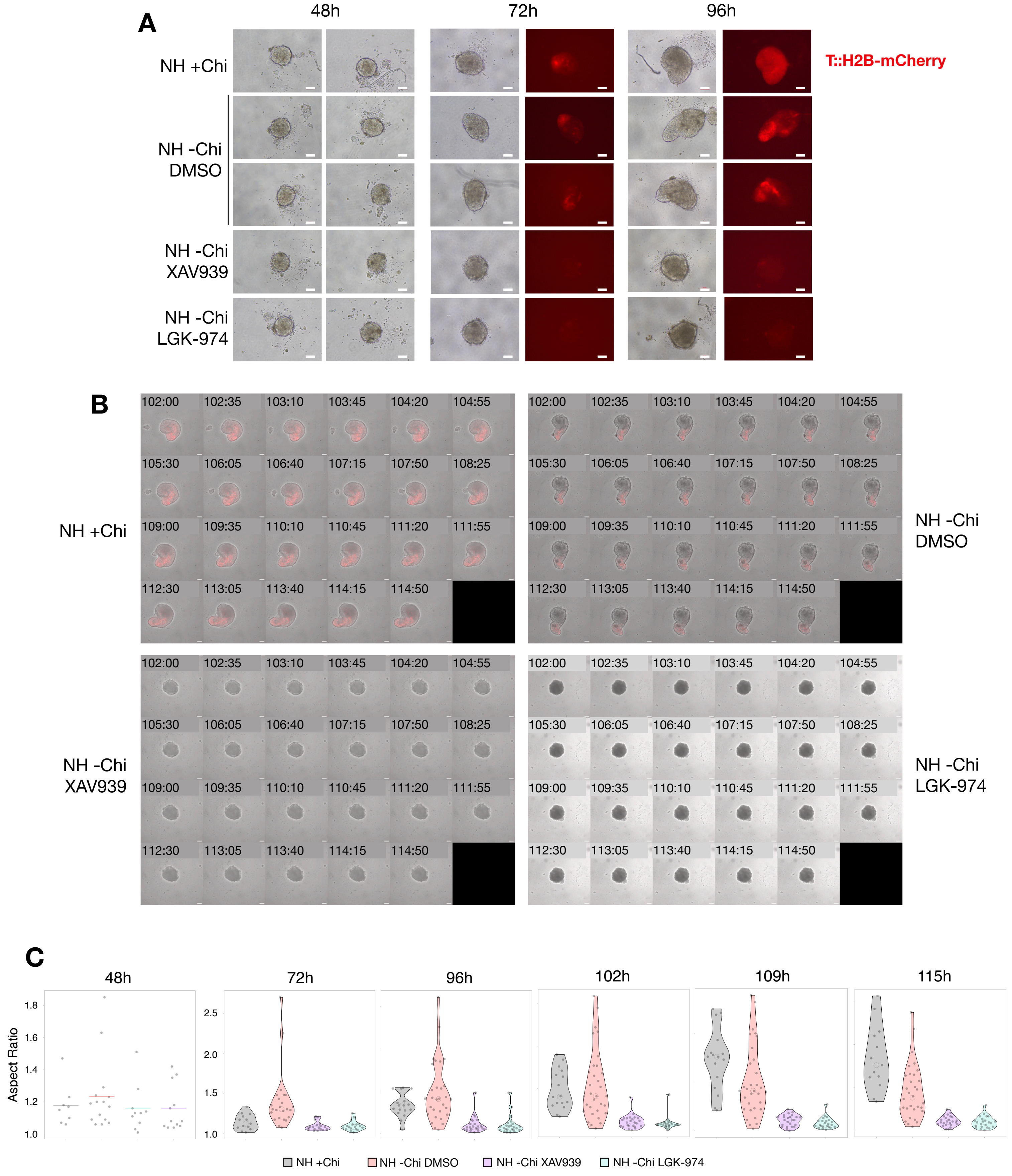

Fig. S5. Investigation of T expression in response to WNT pathway inhibition in spontaneously elongating gastruloids.

A) Expression levels of the T::H2B-mCherry reporter at indicated time points and conditions. Scale bars are 100 μm.

B) Live imaging stills of the T::H2B-mCherry reporter at indicated time points and conditions.

C) Quantification of elongation in the form of aspect ratios. Each dot is a single structure.

Development • Supplementary information

Figure S6

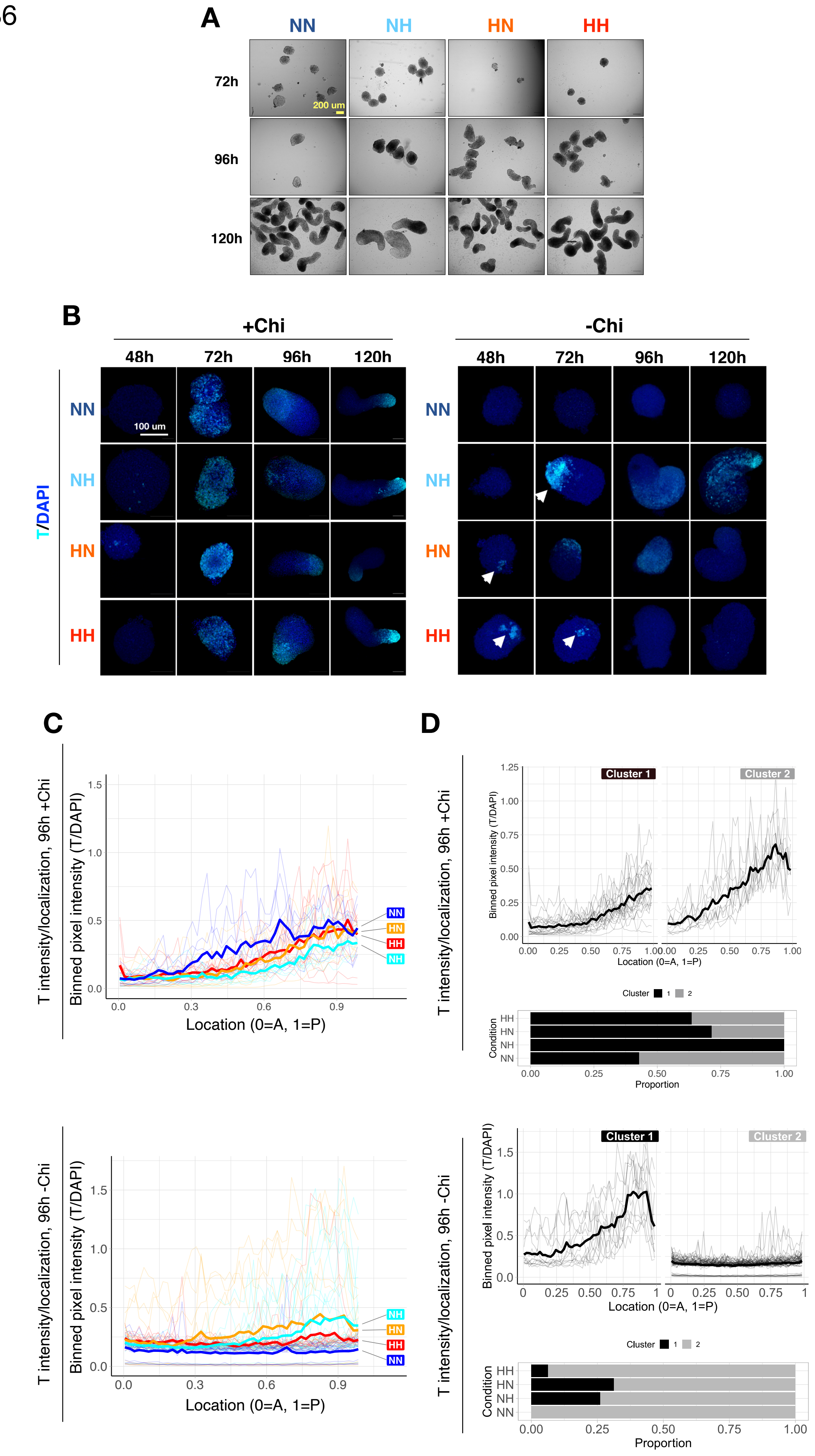

Development • Supplementary information

Fig. S6. Further characterization of T levels in hypoxic +Chi gastruloids exposed to hypoxia.

A) Bright-field images of representative gastruloids at the indicated time points.

B) Fluorescent microscopy images of representative gastruloids at early time points in -Chi and +Chi conditions. Images show a single z-stack. Arrows indicate emergence of T in a subset of cells.

C) Localization of T signal along the anterior-posterior (A-P) axis of gastruloids in each condition at 96h. T signal was binned at 1% length increments along each structure for plotting and normalized to DAPI signal. Thick lines show the mean and thin lines show data from individual structures.

D) K-means clustering of 96h +Chi and -Chi structures with n=2 clusters.

Development • Supplementary information

Figure S7

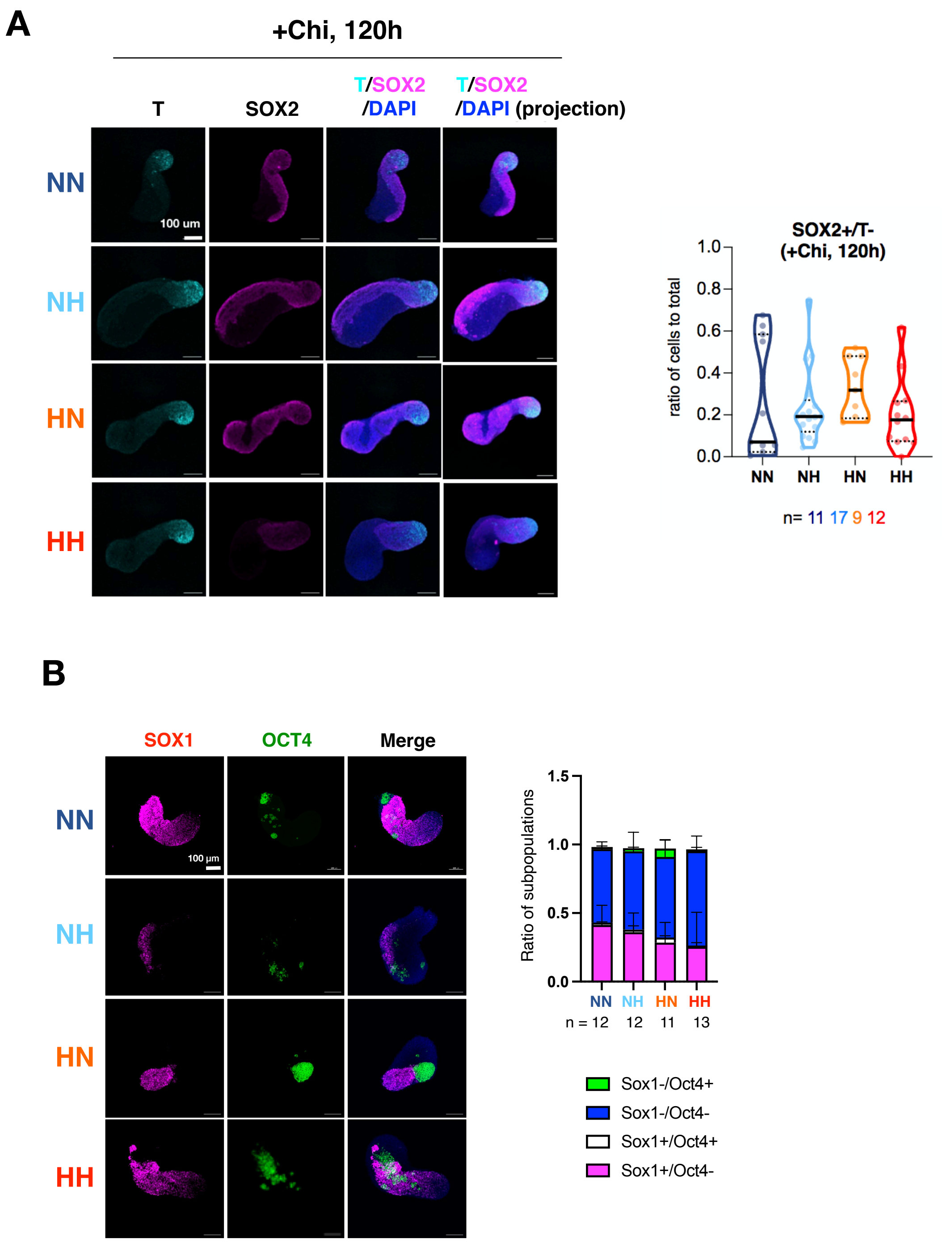

Fig. S7. Characterization of the neural lineage in hypoxic +Chi gastruloids. Representative images (left) and quantification (right) of SOX2 (A) and SOX1 and OCT4 (B) staining in +Chi gastruloids at 120h. n indicates the number of analyzed structures.

Development • Supplementary information

Figure S8

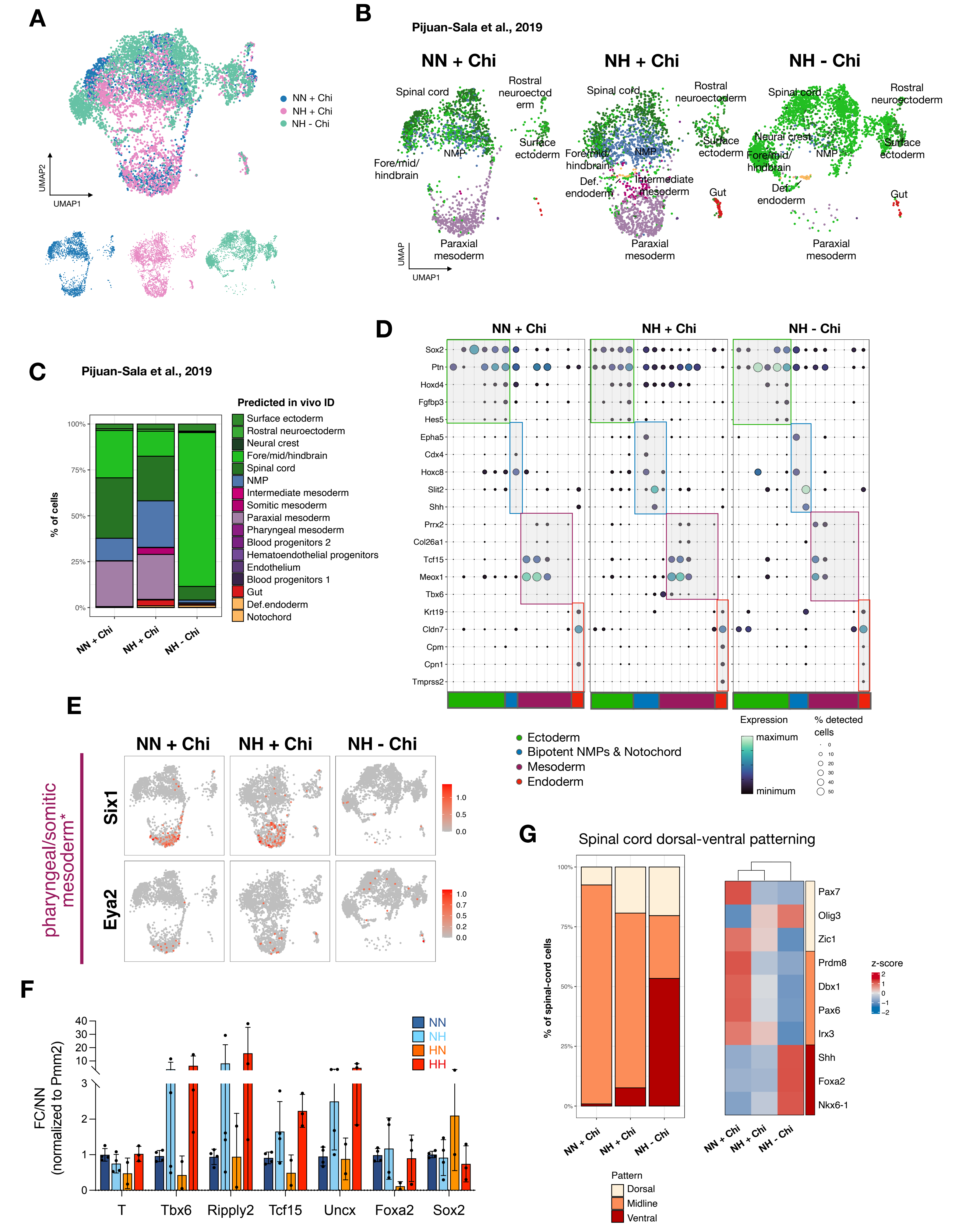

Development • Supplementary information

Fig. S8. Further characterization of scRNA-seq data.

A) Integrated gastruloid UMAP coloured by condition (top) and splitted by condition (bottom).

B) UMAP with Seurat clusters coloured by in vivo predicted ID identified by using reference scRNA-seq atlas from embryonic days E6.5-8.5 (Grosswendt et al., 2020; Pijuan-Sala et al., 2019). See Methods for details. Names of cell states with fewer than 5 cells in a given condition are not listed on the UMAP.

C) Ratio of cells assigned to the indicated in vivo cell state according to the shown reference atlas.

D) Expression levels of top marker genes (Grosswendt et al., 2020) characterizing the in vivo indicated cell states aggregated by germ layer origin.

E) UMAP feature plot coloured by expression of pharyngeal mesoderm marker genes (Grosswendt et al., 2020) at each condition. *Note: these genes have been shown to be expressed as well in somite cell state in (Grifone et al., 2007).

F) Relative expression levels of shown genes in gastruloids at 120h. Data represent FC normalized to Pmm2 and standard deviation for 2-4 biological replicates.

G) Stacked bar plot showing the categorization of spinal cord cells into dorsal-ventral patterning (left), and heatmap showing pseudo-bulk z-score expression values of indicated genes used for the categorization (right).

Development • Supplementary information

Table S1. Full lists of DE genes in ES, TS, and XEN cells

Click here to download Table S1

Table S2. Full lists of GO terms associated with DE genes in ES, TS, and XEN cells

Click here to download Table S2

Table S3. Proteomic profiles of normoxic and hypoxic (d2 and d7) ESCs

Click here to download Table S3

Table S4. Full list of DE proteins in hypoxic ESCs

Click here to download Table S4

Table S5. Full lists of GO terms associated with DE genes at RNA and/or protein levels

Click here to download Table S5

Table S6. List of HIF1α target genes at promoters and distal regions

Click here to download Table S6

Table S7. Full lists of GO terms associated with HIF1α target genes

Click here to download Table S7

Table S8. Full list of HIF1β/α–CATENIN common target promoters and enhancers

Click here to download Table S8

Table S9. Number of gastruloids or structures used for each condition and time point

Click here to download Table S9

Table S10. List of marker genes that we used for neural cell and dorsal-ventral categorizations

Click here to download Table S10

Table S11. Primers and antibodies used in this study

Click here to download Table S11

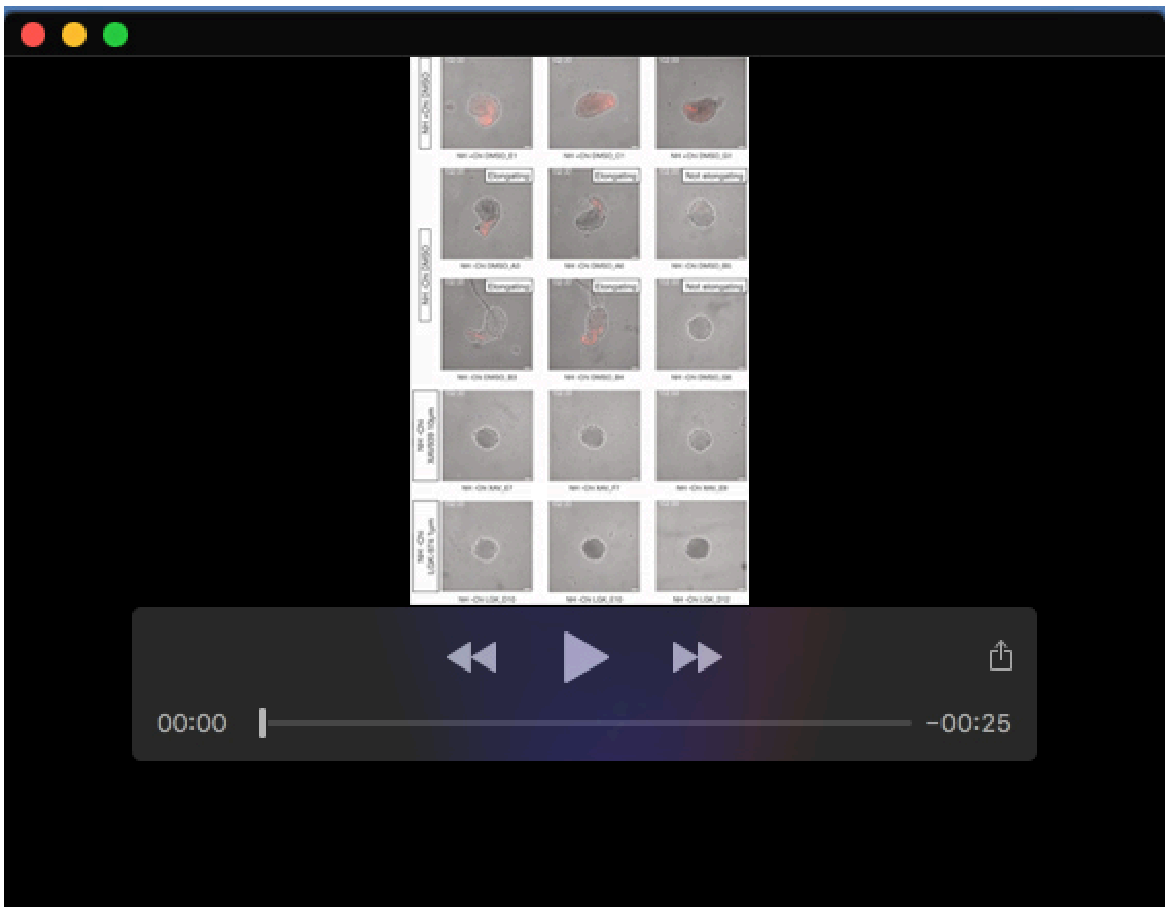

Movie 1. Live imaging of NH -Chi and +Chi gastruloids between 102-115h of differentiation. WNT pathway inhibitors XAV939 and LGK974 were used to test the dependence of elongation on WNT pathay activity. Fluorescence signal shows T::H2B-mCherry reporter activity.
