# Supplementary material for: Hypoxia induces an early primitive streak signature, enhancing spontaneous elongation and lineage representation in gastruloids
Source: Development. 2022 Sep 14;149(20):dev200679. doi: 10.1242/dev.200679 (PMC9578691; doi:10.1242/dev.200679)
Supplement: Supplementary file 1 [file develop-149-200679_review_history.pdf]

Hypoxia induces an early primitive streak signature, enhancing spontaneous elongation and lineage representation in gastruloids

Natalia López-Anguita, Seher Ipek Gassaloglu, Maximilian Stötzel, Adriano Bolondi, Deniz Conkar, Marina Typou, René Buschow, Jesse V. Veenvliet and Aydan Bulut-Karslioglu
DOI: 10.1242/dev.200679

Editor: James Briscoe

Review timeline

Original submission:	25 February 2022
Editorial decision:	11 April 2022
First revision received:	10 June 2022
Editorial decision:	14 July 2022
Second revision received:	26 July 2022
Accepted:	3 August 2022

Original submission

First decision letter

MS ID#: DEVELOP/2022/200679

MS TITLE: Hypoxia induces a transcriptional early primitive streak signature in pluripotent cells enhancing spontaneous elongation and lineage representation in gastruloids

AUTHORS: Natalia Lopez-Anguita, Seher Ipek Gassaloglu, Maximilian Stoetzel, Adriano Bolondi, Marina Typou, Rene Buschow, Jesse Veenvliet, and Aydan Bulut-Karslioglu

Apologies for the delay but I was waiting for a second referee's report. I'm please to say that I have now received all the referees' reports on the above manuscript, and have reached a decision. The referees' comments are appended below, or you can access them online: please go to BenchPress and click on the 'Manuscripts with Decisions' queue in the Author Area.

As you will see, the referees express considerable interest in your work, but have some significant criticisms and recommend a substantial revision of your manuscript before we can consider publication. Reviewer 1 has some helpful suggestions and requests clarifications on a number of points. Reviewer 2 raises more substantial concerns. This reviewer would like a stronger connection made to the in vivo context. While I agree with the reviewer that this is important, in my opinion, the in vitro findings will be of interest to the field on their own. The reviewer makes several suggestions for comparisons with in vivo findings and I would encourage you to discuss these in your manuscript. The reviewer also suggests experiments with ES cells and mice lacking Hif1a. These points are well made. Analysing differentiating ES cells and embryos lacking Hif1a is directly relevant to your study, however, I accept that his is beyond the scope of the current manuscript. Nevertheless, a discussion of these points should be included in a revision. One way to do this would be to include a section on 'Limitation of this study' in your Discussion. This would also allow you to address some of the other concerns Reviewer 2 raises. One issue, which you may already have data to address, is the question of whether the elongation of gastruloids in -CHIR conditions in hypoxia is due to the upregulation of Wnt gene expression. Using a Wnt secretion inhibitor, such as LGK974, would test this.

© 2022. Published by The Company of Biologists under the terms of the Creative Commons Attribution License (https://creativecommons.org/licenses/by/4.0/).

If you are able to revise the manuscript along the lines suggested, I will be happy receive a revised version of the manuscript. Your revised paper will be re-reviewed by one or more of the original referees, and acceptance of your manuscript will depend on your addressing satisfactorily the reviewers' major concerns. Please also note that Development will normally permit only one round of major revision. If it would be helpful, you are welcome to contact us to discuss your revision in greater detail. Please send us a point-by-point response indicating your plans for addressing the referee's comments, and we will look over this and provide further guidance.

Please attend to all of the reviewers' comments and ensure that you clearly highlight all changes made in the revised manuscript. Please avoid using 'Tracked changes' in Word files as these are lost in PDF conversion. I should be grateful if you would also provide a point-by-point response detailing how you have dealt with the points raised by the reviewers in the 'Response to Reviewers' box. If you do not agree with any of their criticisms or suggestions please explain clearly why this is so.

Reviewer 1

Advance summary and potential significance to field

The present manuscript is a comprenhensive study on how more physiological oxygen levels and hypoxia/normoxia transitions might affect mouse development. The authors show that subtle transcriptional differences (many of which not detected by MS) in WNT pathway can lead to measurable effects in an organoid context.
Although some of the reported hypoxia results have been described previously their effects on gastruloids are novel and have the potential to change how in vitro models of early development are generated. As such, I am recommmending it for publication.

Comments for the author

Comments:
- Information and images (in past rebuttal) on cell density in bulk RNA-seq should be included.
- In general, HN vs NH should be further discussed. The results do not seem to match with the expected in vivo case.
- Line 273: "NH and HN -Chi aggregates (~30% of all structures in NH or HN vs 0% in NN condition) (Figure 4F)" Not sure where in the figure should i see this.
Also, cluster0/1 distributions look extremely identical.
- Line 276: "compare distribution of NH vs HN -Chi at 120h" - these are not convincing.
- Figure 4B: clarify if by stack vs 3D the authors mean single-z plane vs MIP ?
- Quantification of western blot in Fig. S3C used to state that B-cat expression isn't altered.
- Quantification performed in S6A should be clarified. This section in the Methods is rather vague.. the authors claim > 10 million single nuclei were analysed but we have no information on how reliable this data is or if it accurately represents the images.
- Figure 7H. Dark and light green shades marking forebrain anbd spinal cord.
Markers should be clarified and also the colors appear different in the stacked bar plot below.

Reviewer 2

Advance summary and potential significance to field

The study by Anguita et al. investigates the response of mouse embryonic stem cells and gastruloids to hypoxia. They find that hypoxia induces the expected upregulation of glycolysis and also leads to gene expression changes, including alteration in mRNA levels of Wnt signaling targets. They conclude that the change in glycolysis is not causally related to the changes in gene expression. Most notably, perhaps, is their finding that in gastruloids, hypoxic conditions are corelated with elongation phenotypes, even though no additional stimulus with CHIR (a pleiotropic GSK inhibitor) is given. These elongated structures lack, in contrast to control gastruloids, mesoderm. The authors put forward a model in " which hypoxia directly induces endoderm and notochord, and in turn notochord together with exogenous WNT activation may enhance mesodermal progenitors and derivatives."

© 2022. Published by The Company of Biologists under the terms of the Creative Commons Attribution License (https://creativecommons.org/licenses/by/4.0/).

Comments for the author

As I see from their responses to previous comments they received, the authors have revised and improved their manuscript. They present a large amount of experiments and data, there is no question that interesting observations are made.
However, in my view there are main issues, some of these had been raised previously, listed below. My general concern is the following: It is to be expected that hypoxia changes the biology of any system, impacting numerous signaling and metabolic activities simultaneously. In addition, the response depends highly on the duration, dose and context of the hypoxia treatment.
The key general question then is, what do we learn from observing these responses in in vitro systems, i.e. to which extend and how do these relate to the physiological context? It is the motivation of the authors to consider the in vivo context, as they introduce, rightly, that the mouse embryo is facing changing, also hypoxic, environments. However, important previous findings, the integration/link to in vivo models and also key controls (see below) are missing. I am not convinced their approach and data allows to make a reference to these fundamental questions that motivated the study.
(1) From a mechanistic point of view, the key question is how hypoxia is exerting its effect(s). It is clear from previous studies that a major mediator is Hif1alpha, but also Hif-independent mechanisms are described (see Damert et al. 1997). Hence, the role and requirement of Hif1alpha needs to be tested, directly.
Knock-out ES-cells have been generated more than 20 years ago, there are several seminal papers cited literally thousands of times (see Iyer et al. 1997., Ryan et al., 1998).
This approach was essential to test the potential of these cells in teratoma assays. In addition, an importantly, this approach also identified Hif1 dependent and Hif1-independent responses to Hypoxia. I think that for any study using ES-cells to address the role of Hypoxia, the use of these KO ES-cells is mandatory. In their response to the reviewer the authors describe their attempts to generate these lines themselves, without success. Previous reports demonstrate it is technically/biologically feasible. I would encourage to contact these groups or repositories. In my view, it is not possible to address, as the authors aim, the “ molecular effects of acute and prolonged hypoxia on transcriptional networks and stem cell identities of embryonic and extraembryonic stem cells.” without the inclusions of such a well-defined, molecular functional approach/control.

(2) In my view, the findings need to be linked and compared to the previous results obtained in vivo, such as Hif1alpha knock out embryos. Previous reports have shown that Hif1alpha KO embryos develop beyond gastrulation stages, i.e. form a body axis, and anomalies arise/become visible on day 8 onwards (see Iyer et al. 1997., Ryan et al., 1998). The Hif1alpha KO model is available from repositories (see for example JAX, https://www.jax.org/strain/007561). Inclusion of this previous information and also use of the available mouse model is needed to connect the findings made in vitro to a physiological context.
(3) The role and effect of hypoxia in the culture of mouse embryos is being discussed since more than 50 years (see for instance, Tam and Snow 1980). More recently, a detailed study (cited in this manuscript) has addressed the outcome of different oxygen-levels during the culture of gastrulation stage mouse embryos (Aguilera-Castrejon et al. 2021, see their Supplementary Figure 2 with very comprehensive analysis of different oxygen-levels). Can this data/approach be linked to the findings of this current work, i.e. is differentiation affected, quantitatively, by culturing the embryos in different oxygen levels? Would the outcome be consistent with the results obtained using gastruloids?
(4) I agree with reviewer 3 that one of the most interesting findings is the elongation of gastruloids in hypoxia, even in the -CHIR condition. However, it remains unclear what the underlying mechanism is and how and whether it relates to the findings made in ES-cells, i.e. a modest upregulation of Wnt-target genes. For instance, is the effect of hypoxia in NH-CHIR gastruloids dependent on Wnt-signaling at all?
(5) Related, for how long is hypoxia treatment needed to induce this effect? Are alternative signaling pathways triggered by hypoxia, also explaining why these gastruloids (NH-CHIR) seem to lack mesoderm (which forms under the influence of Wnt-signaling)?
(6) How do the findings in ES-cells account for the finding in gastruloids, i.e. that hypoxia) is showing a greater effect during the second stage of the differentiation protocol (NH-CHIR)? Again, the connection to a physiological setting is needed here and could be achieved by integrating

© 2022. Published by The Company of Biologists under the terms of the Creative Commons Attribution License (https://creativecommons.org/licenses/by/4.0/).

mouse embryo culture, see point 3 above. Again, I see the risk of describing and studying a highly context specific outcome, without clear relevance to physiological context.
(7) I will not comment in detail on the metabolic/glycolytic perturbations, data and conclusions - one more general comment: It is not possible, in my view, “to dissect the influence of glycolysis” by a single perturbation, very indirectly influencing glycolysis via an inhibition of oxphos, as the authors do. While the increase in lactate indicates that glycolytic flux was changed, a much more direct perturbation and detailed view is needed. The inhibition of oxphos, which anyway should not be highly active given the context of hypoxia, will have different effects compared to a more direct activation of glycolysis, for instance with different amount of glucose, or by genetic means to activate flux at defined positions within the glycoltyic pathway, taking into account the branching points to other, connected pathways, i.e. PPP, Hexosaminepathway, One-carbon metabolism etc. The outcome of these different ways to perturb 'glycolysis' can be expected to be distinct, based on previous studies, which could have important implications for the conclusions.

First revision

Author response to reviewers' comments

Reviewer #1
Advance Summary and Potential Significance to Field:
The present manuscript is a comprehensive study on how more physiological oxygen levels and hypoxia/normoxia transitions might affect mouse development. The authors show that subtle transcriptional differences (many of which not detected by MS) in WNT pathway can lead to measurable effects in an organoid context. Although some of the reported hypoxia results have been described previously, their effects on gastruloids are novel and have the potential to change how in vitro models of early development are generated. As such, I am recommending it for publication.

Reviewer 1 Comments for the Author:
- Information and images (in past rebuttal) on cell density in bulk RNA-seq should be included.

We now included the relevant image in Figure S1D and inserted the following sentence in to the main text: “Cells were cultured at similarly low densities across conditions to eliminate colony size as a barrier to oxygen diffusion (Figure S1D).”

- In general, HN vs NH should be further discussed. The results do not seem to match with the expected in vivo case.

We show that spontaneous elongation also occurs in HN -Chi, is however more efficient in NH -Chi condition. This may be due to higher levels of WNT induction at NH -Chi structures at 72h and therefore timing of peak WNT activity as well as its interplay with SOX2. A recent publication showed that the levels of SOX2 shape WNT activity (Blassberg, R., Patel, H., Watson, T. *et al.* Sox2 levels regulate the chromatin occupancy of WNT mediators in epiblast progenitors responsible for vertebrate body formation. *Nat Cell Biol* **24**, 633–644 (2022), https://doi.org/10.1038/s41556-022- 00910-2). The precise amounts of these two regulators are likely dependent on culture conditions (ESC vs differentiation media) and shape the cellular profile in NH -Chi structures, e.g. by promoting WNT-driven differentiation upon exit from pluripotency. To address this and other key points, we now included a Limitations of the Study section after the Discussion.

- Line 273: "NH and HN -Chi aggregates (~30% of all structures in NH or HN vs 0% in NN condition) (Figure 4F)" Not sure where in the figure should i see this. The ~30% rate of formation of a T-positive pole is shown in Figure 4F. In this figure we focused on NH and HN conditions and thus the negative control NN -Chi condition is not shown. Figure S4D shows the 0% T-positive rate of the NN -Chi condition.

Also, cluster0/1 distributions look extremely identical.

We assume the Reviewer means the proportion of clusters 1 and 2 in NH vs HN -Chi conditions (Figure 4F). These proportions do indeed look very similar, and a similar distribution is also seen

© 2022. Published by The Company of Biologists under the terms of the Creative Commons Attribution License (https://creativecommons.org/licenses/by/4.0/).

at 96h in Figure S4D. Thus, NH and HN conditions overall show similar proportions of T positivity, which are much higher than NN and HH -Chi conditions.

- Line 276: "compare distribution of NH vs HN -Chi at 120h" - these are not convincing.

We revised the sentence as: “However, T expression level is lower in HN -Chi structures compared to NH -Chi (cluster 1 in each condition, Figure 4F), *which might explain the better elongation of NH -Chi structures at 120h* (Figure 4D).”

- Figure 4B: clarify if by stack vs 3D the authors mean single-z plane vs MIP ?

Yes, we meant single z-plane and 3D projection comprising of all stacks. To make this clearer, we now labeled these as *z-plane* and *projection*.

- Quantification of western blot in Fig. S3C used to state that B-cat expression isn't altered.

We could quantify the western blot, however believe that western blotting does not provide precise enough data to be quantified. In addition, the blot in Figure S3C shows the total levels of the protein, which comprises of phosphorylated and non-phosphorylated subsets. A more precise quantification of the phosphorylated protein would be necessary to follow up on this point.

- Quantification performed in S6A should be clarified. This section in the Methods is rather vague.. the authors claim > 10 million single nuclei were analysed but we have no information on how reliable this data is or if it accurately represents the images.

We analyzed all stained structures without excluding any of them. The numbers of structures analyzed per condition are provided in Table S9. The masking of individual cells by DAPI is described in the methods. This type of masking is quite standard and therefore we deem is too detailed to show the actual masking in the paper. For the Reviewer we provide here a snapshot of the DAPI masking in one of the structures.

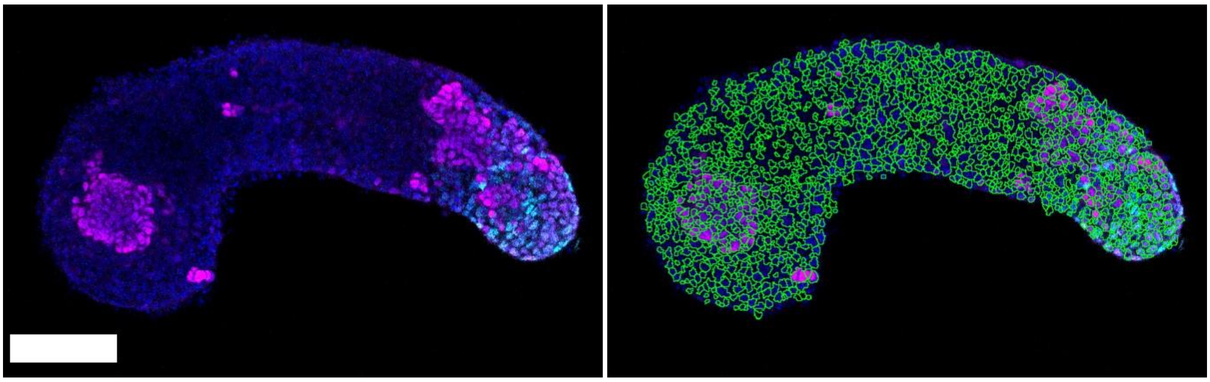

- Figure 7H. Dark and light green shades marking forebrain and spinal cord. Markers should be clarified and also the colors appear different in the stacked bar plot below.

If the Reviewer means that the green shades are not distinguishable: We selected distinct light and dark green shades that are visible in print and on screen. We did not assign a different color to either category because they both belong to the ectodermal lineage, and this lineage is shown in green in Figure 7A. We now added a supplementary table (Table S10) showing the markers used for the reclassification.

The colors in the stacked bar plot are deliberately different. Since we reclassify the annotated forebrain and spinal cord cells in a separate analysis, we intended to avoid misleading the reader into thinking that the classification is based on the previous annotations. To help readers to understand this new re-classification, we use different colors.

Reviewer #2

Advance Summary and Potential Significance to Field:

The study by Anguita et al. investigates the response of mouse embryonic stem cells and gastruloids to hypoxia. They find that hypoxia induces the expected upregulation of glycolysis and also leads to gene expression changes, including alteration in mRNA levels of Wnt signaling targets. They conclude that the change in glycolysis is not causally related to the changes in gene

© 2022. Published by The Company of Biologists under the terms of the Creative Commons Attribution License (https://creativecommons.org/licenses/by/4.0/).

expression. Most notably, perhaps, is their finding that in gastruloids, hypoxic conditions are corelated with elongation phenotypes, even though no additional stimulus with CHIR (a pleiotropic GSK inhibitor) is given. These elongated structures lack, in contrast to control gastruloids, mesoderm. The authors put forward a model in " which hypoxia directly induces endoderm and notochord, and in turn notochord together with exogenous WNT activation may enhance mesodermal progenitors and derivatives."

Comments for the Author:
As I see from their responses to previous comments they received, the authors have revised and improved their manuscript. They present a large amount of experiments and data, there is no question that interesting observations are made.

However, in my view there are main issues, some of these had been raised previously, listed below.

My general concern is the following: It is to be expected that hypoxia changes the biology of any system, impacting numerous signaling and metabolic activities simultaneously. In addition, the response depends highly on the duration, dose and context of the hypoxia treatment.
The key general question then is, what do we learn from observing these responses in in vitro systems, i.e. to which extend and how do these relate to the physiological context? It is the motivation of the authors to consider the in vivo context, as they introduce, rightly, that the mouse embryo is facing changing, also hypoxic, environments. However, important previous findings, the integration/link to in vivo models and also key controls (see below) are missing. I am not convinced their approach and data allows to make a reference to these fundamental questions that motivated the study.
We thank the Reviewer for their perspective. Although it is true that direct experimental comparisons to the embryo are missing, we note that this is not the primary motivation of this manuscript. We aimed to dissect the hypoxia response in in vitro ESC and gastruloid models because these models provide more controlled conditions for dissection and manipulation of the timing and duration of hypoxia and its components. In addition, we believe the gastruloid model provides an appropriate platform to investigate cell type specification events, uncomplicated by interference with maternal factors, vascularization defects, etc. One of our main aims is to harness gene-environment interactions to optimize in vitro models of development. Within this context we implement hypoxia into the gastruloid protocol and show increased tissue complexity in +Chi and spontaneous elongation in -Chi conditions.

(1) From a mechanistic point of view, the key question is how hypoxia is exerting its effect(s). It is clear from previous studies that a major mediator is Hif1alpha, but also Hif-independent mechanisms are described (see Damert et al. 1997). Hence, the role and requirement of Hif1alpha needs to be tested, directly. Knock-out ES-cells have been generated more than 20 years ago, there are several seminal papers cited literally thousands of times (see Iyer et al. 1997., Ryan et al., 1998). This approach was essential to test the potential of these cells in teratoma assays. In addition, an importantly, this approach also identified Hif1 dependent and Hif1-independent responses to Hypoxia. I think that for any study using ES-cells to address the role of Hypoxia, the use of these KO ES-cells is mandatory. In their response to the reviewer the authors describe their attempts to generate these lines themselves, without success. Previous reports demonstrate it is technically/biologically feasible. I would encourage to contact these groups or repositories. In my view, it is not possible to address, as the authors aim, the " molecular effects of acute and prolonged hypoxia on transcriptional networks and stem cell identities of embryonic and extraembryonic stem cells." without the inclusions of such a well-defined, molecular functional approach/control.
We agree with the reviewer that the KO model is essential to test Hif1a dependency, however we did not claim the dependency of our findings on Hif1a, but rather showed that Hif1a activation is sufficient to replicate the effects. Nevertheless, we did indeed contact and receive Hif1a KO cells from other researchers, which had mycoplasma contamination and could not be used. We now include a 'Limitations of the study' section to address this and other key points.

(2) In my view, the findings need to be linked and compared to the previous results obtained in vivo, such as Hif1alpha knock out embryos. Previous reports have shown that Hif1alpha KO embryos develop beyond gastrulation stages, i.e. form a body axis, and anomalies arise/become

© 2022. Published by The Company of Biologists under the terms of the Creative Commons Attribution License (https://creativecommons.org/licenses/by/4.0/).

visible on day 8 onwards (see Iyer et al. 1997., Ryan et al., 1998). The Hif1alpha KO model is available from repositories (see for example JAX, https://www.jax.org/strain/007561). Inclusion of this previous information and also use of the available mouse model is needed to connect the findings made in vitro to a physiological context.
We thank the Reviewer for the suggestion. The Hif1a KO embryos show defects in somites and the neural tube/fold, which are in agreement with our results in Figure 7 of the beneficial effects of hypoxia on enhancing the somite and notochord signatures. However, the Hif1a KO mouse embryos also show severe vascularization defects, complicating the interpretation of their phenotype. Although the body axis is formed and the Hif1a KO embryos survive up until midgestation, their defects may arise at a much earlier time point, and even at gastrulation. Detailed lineage-tracing experiments are necessary to investigate this possibility, which we deem outside the scope of this study.
The Hif1a KO models were cited in an earlier version of the manuscript, which we later removed due to space limitations. We now include a 'Limitations of the study' section to address this and other key points.

(3) The role and effect of hypoxia in the culture of mouse embryos is being discussed since more than 50 years (see for instance, Tam and Snow 1980). More recently, a detailed study (cited in this manuscript) has addressed the outcome of different oxygen-levels during the culture of gastrulation stage mouse embryos (Aguilera-Castrejon et al. 2021, see their Supplementary Figure 2 with very comprehensive analysis of different oxygen-levels). Can this data/approach be linked to the findings of this current work, i.e. is differentiation affected, quantitatively, by culturing the embryos in different oxygen levels? Would the outcome be consistent with the results obtained using gastruloids?
We agree that the mentioned study provides a great comparison to embryo development, however we note that the role of hypoxia cannot be clearly dissected in the mentioned system due to the culture of embryos in media that contains a very high percentage of rat serum, and therefore rich in growth factors and nutrients. In our opinion, in vitro stem cell models provide a more controlled environment for pathway dissection and for investigation of the flexibility and boundaries of cellular decision making. Nevertheless, Aguilera-Castrejon et al. showed that hypoxia exposure within the first days of the in vitro embryo culture is beneficial and chose to gradually increase oxygen concentration from 5 to 13 to 18 to 21% until day 4. Although the mentioned paper does not present any data on the cell type composition of embryos cultured under different oxygen concentrations, the results are overall in agreement with our findings that hypoxia is a beneficial microenvironmental factor during the earliest phases of differentiation.

(4) I agree with reviewer 3 that one of the most interesting findings is the elongation of gastruloids in hypoxia, even in the -CHIR condition. However, it remains unclear what the underlying mechanism is and how and whether it relates to the findings made in ES-cells, i.e. a modest upregulation of Wnt-target genes. For instance, is the effect of hypoxia in NH-CHIR gastruloids dependent on Wnt- signaling at all?
To answer this question, we performed extensive new experiments and tested elongation of NH - Chi structures in the absence or presence of two different WNT pathway inhibitors: the PORCN inhibitor LGK974, which blocks WNT secretion; and the Tankyrase inhibitor XAV939, which promotes degradation of b-catenin. We assessed WNT pathway activity both via RT-qPCR of its downstream targets T and Eomes and by live imaging of the T reporter previously used in the paper (T::H2B-mCherry). In addition to gene expression, spontaneous elongation was quantified via morphometric analysis of bright field images of the generated structures. Spontaneous elongation is lost upon treatment with both WNT inhibitors in both backgrounds (wt and reporter) . As expected, WNT downstream targets T and Eomes and the T reporter could not be activated. We include this new dataset in Figures S4 and S5 and explain in the text as: '*Hypoxia-induced spontaneous elongation is WNT pathway-dependent, as treatment with the PORCN inhibitor LGK-974 and the Tankyrase inhibitor XAV939 during hypoxia exposure results in loss of spontaneous elongation as well as induction of T and Eomes (Figs S4C, S5A-C, Movie 1).* '

(5) Related, for how long is hypoxia treatment needed to induce this effect?
Our ESC results show that T, Eomes and Wnt3 upregulation is first observed on day 3 (Figure 2A) and gradually increase until day 16 (Figure S2E). Similarly, T expression is first seen in the NH-Chi structures at 72 hours post aggregation (Figure S4B). Based on these results, hypoxia exposure is likely needed for a minimum of three days to induce WNT pathway activity. We did

© 2022. Published by The Company of Biologists under the terms of the Creative Commons Attribution License (https://creativecommons.org/licenses/by/4.0/).

not yet test whether a transient three day exposure is sufficient to induce elongation in NH -Chi structures. A detailed analysis of hypoxia exposure duration, period as well as oxygen and Chiron titrations are planned within the scope of a follow-up study.
Are alternative signaling pathways triggered by hypoxia, also explaining why these gastruloids (NH-CHIR) seem to lack mesoderm (which forms under the influence of Wnt-signaling)?
Alternative signaling pathways may be triggered, however did not come up in our gene ontology analysis. Several WNT, BMP and FGF proteins are induced in hypoxic ES cells (Figure S2B). Regarding why NH -Chi structures lack mesoderm, we think that this is either due to insufficient WNT activity or altered activity of the neuromesodermal progenitors in favor of the neural lineage. Since these structures are not treated with Chiron, cells are not ubiquitously primed towards the mesodermal lineage and thus NMPs might preferably generate the neural lineage. A recent publication showed that the levels of SOX2 shape WNT activity, and the precise amounts of these two regulators likely shape the cellular profile in NH -Chi structures as well (Blassberg, R., Patel, H., Watson, T. *et al.* Sox2 levels regulate the chromatin occupancy of WNT mediators in epiblast progenitors responsible for vertebrate body formation. *Nat Cell Biol* **24,** 633–644 (2022), https://doi.org/10.1038/s41556-022-00910-2).

(6) How do the findings in ES-cells account for the finding in gastruloids, i.e. that hypoxia) is showing a greater effect during the second stage of the differentiation protocol (NH-CHIR)? Again, the connection to a physiological setting is needed here and could be achieved by integrating mouse embryo culture, see point 3 above. Again, I see the risk of describing and studying a highly context specific outcome, without clear relevance to physiological context.
We agree that a thorough comparison to in vitro embryo culture would help draw parallels between the two systems, however as mentioned in point 3, the culture conditions of embryos are very different than of gastruloids. It is our motivation to harness hypoxia as a guiding factor to achieve a more complex and balanced cell type landscape in gastruloids, rather than reproducing the in vivo setting exactly as it is.
Regarding the effects of hypoxia exposure on ES cells vs during differentiation: Our results in both cases are largely consistent, with hypoxia inducing the WNT pathway as well as an endoderm signature in both cases. The timing is also consistent (please see point 5 above). We show that spontaneous elongation also occurs in HN -Chi, is however more efficient in NH -Chi condition. This may be due to higher levels of WNT induction at NH -Chi structures at 72h and therefore timing of peak WNT activity as well as its interplay with SOX2, which is likely the reason of the permissiveness of the differentiation media to induction of primitive streak genes at the protein level.

(7) I will not comment in detail on the metabolic/glycolytic perturbations, data and conclusions - one more general comment: It is not possible, in my view, “to dissect the influence of glycolysis” by a single perturbation, very indirectly influencing glycolysis via an inhibition of oxphos, as the authors do. While the increase in lactate indicates that glycolytic flux was changed, a much more direct perturbation and detailed view is needed. The inhibition of oxphos, which anyway should not be highly active given the context of hypoxia, will have different effects compared to a more direct activation of glycolysis, for instance with different amount of glucose, or by genetic means to activate flux at defined positions within the glycoltyic pathway, taking into account the branching points to other, connected pathways, i.e. PPP, Hexosaminepathway, One-carbon metabolism etc. The outcome of these different ways to perturb 'glycolysis' can be expected to be distinct, based on previous studies, which could have important implications for the conclusions.
We thank the reviewer for these insights. Our expectation, as the Reviewer’s is, was that hypoxic cells would not employ oxphos anyway, however Figure 2F shows that inhibition of oxphos has a dynamic effect on even hypoxic cells. Our data show that a global shift in glycolytic flux does not replicate the hypoxia-induced expression of primitive streak genes, although we agree that a much more detailed analysis of subcomponents and branches of the glycolysis pathway and connected pathways would be even more informative.

© 2022. Published by The Company of Biologists under the terms of the Creative Commons Attribution License (https://creativecommons.org/licenses/by/4.0/).

Second decision letter

MS ID#: DEVELOP/2022/200679

MS TITLE: Hypoxia induces an early primitive streak signature, enhancing spontaneous elongation and lineage representation in gastruloids

AUTHORS: Natalia Lopez-Anguita, Seher Ipek Gassaloglu, Maximilian Stoetzel, Adriano Bolondi, Deniz Conkar, Marina Typou, Rene Buschow, Jesse Veenvliet, and Aydan Bulut-Karslioglu

I have now received all the referees reports on the above manuscript, and have reached a decision. The referees' comments are appended below, or you can access them online: please go to BenchPress and click on the 'Manuscripts with Decisions' queue in the Author Area.

The overall evaluation is positive and we would like to publish a revised manuscript in Development, provided that the referees' comments can be satisfactorily addressed. Reviewer 2 remains concerned about how some of your interpretations and conclusions are worded. I think these issues can be dealt with by rephrasing relevant paragraphs and ensuring that your intended meaning is clarified by additional explanation, where necessary. Please attend to all of the reviewers' comments in your revised manuscript and detail them in your point-by-point response. If you do not agree with any of their criticisms or suggestions explain clearly why this is so. If it would be helpful, you are welcome to contact us to discuss your revision in greater detail. Please send us a point-by-point response indicating your plans for addressing the referee's comments, and we will look over this and provide further guidance.

Reviewer 1

Advance summary and potential significance to field

Thank you for all the clarifications.

Comments for the author

One last suggestion is to make the legend of Fig.7H more complete to explain its diagram, i.e. that forebrain and spinal cord cells were grouped and re-
classified.

Reviewer 2

Advance summary and potential significance to field

The authors have addressed some of my concerns, adding new experiments that show the Wnt-dependency of NH-CHIR gastruloids and they have included a new paragraph "Limitations of the study", which somewhat added to increase clarity (but see below). I find this revised manuscript improved. However, several of the major points I raised remain unadressed, I will only focus on three points:

1) "HIF1α mediates the transcriptional priming in hypoxic ESCs"

I remain of the opinion that in order to make this statement, the functional requirement for Hif1a needs to be directly tested. While the authors answered " "we did not claim the dependency of our findings on Hif1a", I think that above conclusion does imply exactly this to the reader, i.e. the effect is mediated and hence dependent on Hif1a. Therefore, in my view, testing whether the effect requires Hif1a is critical. I also think that since the authors use the in vitro system as it provides the means to have a more controllable setting, my insistence of having this additional experiment added is not just scientifically warranted, but also reasonable.

2) Relevance of findings to in vivo development

© 2022. Published by The Company of Biologists under the terms of the Creative Commons Attribution License (https://creativecommons.org/licenses/by/4.0/).

“Although it is true that direct experimental comparisons to the embryo are missing, we note that this is not the primary motivation of this manuscript.” and they add that their goal is to “to optimize in vitro models of development”.
To me their reply is somewhat confusing. While the in vitro models can be used to learn about development, they can also serve other purposes, such as the engineering of certain cell types for applications. However, since they do use it to model development, I think that a closer comparison to the in vivo situation, for instance by referring carefully to the Hif1a KO embryos is needed.

In this regard, the authors now state that “Hif1α KO embryos show defects in somites and the neural tube/fold, which are in agreement with our results of the beneficial effects of hypoxia on enhancing the somite and notochord signatures (Fig. 7). However, the Hif1α KO mouse embryos also show severe vascularization and placental defects, complicating the interpretation of their phenotype (Kozak et al., 1997).”
This statement needs to be refined, I think. As far as I know, there is no vascularization phenotype described at day 8.5dpc in Hif1a KO embryos, this phenotype manifests later. Moreover, from the in vivo KO data, there is no evidence that germ layer specification or gastrulation is affected. To conclude that the in vitro expression analysis (Fig.7) is agreeing with the KO in vivo phenotype appears therefore misleading. Again, while this can certainly be addressed by rephrasing this paragraph, it re-enforces the importance to have clear functional evidence for the role of Hif1 for the observed phenotype, in vitro. Once this is clarified, only then the comparison to the in vivo Hif1a KO phenotype is actually relevant.

3) The authors have not addressed my concern about their conclusions on the role of glycolysis. “The metabolic shift to glycolysis does not contribute to the hypoxia-mediated early primitive streak signature” is in my view a conclusion that is not supported sufficiently by their data. As I outlined previously, the issue I see is that the authors do not perturb glycolysis directly. They only apply a perturbation strategy using the mitochondrial inhibitor FCCP (and combine this treatment with hypoxia treatment, complicating the expected impact on glycolysis further). How and if this affects glycolysis is unclear and not investigated, beyond measurement of lactate (which can, however, be altered for several reasons). Along this line, it is of note that upon FCCP treatment, Pfkfb3 expression is not increased (see Figure 2 G), even though it is known that increased flux will lead to increased Pfkfb3 expression. Without doubt, more direct manipulation of glycolytic flux is needed and feasible in this simplified in vitro setting.

Comments for the author

See above.

Second revision

<u>Author response to reviewers' comments</u>

Reviewer 1 Advance Summary and Potential Significance to Field:
Thank you for all the clarifications.

Reviewer 1 Comments for the Author:
One last suggestion is to make the legend of Fig.7H more complete to explain its diagram, i.e. that forebrain and spinal cord cells were grouped and re-classified.

We added the following explanation to the legend of Figure 7H: ‘The annotated forebrain and spinal cord cells from (A) have been regrouped and reclassified based on the shown criteria.’

Reviewer 2 Advance Summary and Potential Significance to Field:
The authors have addressed some of my concerns, adding new experiments that show the Wnt-dependency of NH-CHIR gastruloids and they have included a new paragraph “Limitations of the study”, which somewhat added to increase clarity (but see below). I find this revised manuscript

© 2022. Published by The Company of Biologists under the terms of the Creative Commons Attribution License (https://creativecommons.org/licenses/by/4.0/).

improved. However, several of the major points I raised remain unadressed, I will only focus on three points:
1)“HIF1α mediates the transcriptional priming in hypoxic ESCs”
I remain of the opinion that in order to make this statement, the functional requirement for Hif1a needs to be directly tested. While the authors answered “ "we did not claim the dependency of our findings on Hif1a”, I think that above conclusion does imply exactly this to the reader, i.e. the effect is mediated and hence dependent on Hif1a. Therefore, in my view, testing whether the effect requires Hif1a is critical. I also think that since the authors use the in vitro system as it provides the means to have a more controllable setting, my insistence of having this additional experiment added is not just scientifically warranted, but also reasonable.

Here we respectfully disagree with the Reviewer. It has been shown on numerous occasions in the literature that a factor that is sufficient may not be necessary, or that gain of function vs loss of function phenotypes may be independent of each other. Here we show that chemical induction of HIF1a in normoxia is sufficient to replicate our findings. This does not mean that HIF1a is required and we do not imply this. To avoid further confusion we now revised the relevant conclusion ‘Chemical activation of HIF1a replicates WNT pathway induction by hypoxia’

2) Relevance of findings to in vivo development

“Although it is true that direct experimental comparisons to the embryo are missing, we note that this is not the primary motivation of this manuscript.” and they add that their goal is to “to optimize in vitro models of development”.
To me their reply is somewhat confusing. While the in vitro models can be used to learn about development, they can also serve other purposes, such as the engineering of certain cell types for applications. However, since they do use it to model development, I think that a closer comparison to the in vivo situation, for instance by referring carefully to the Hif1a KO embryos is needed.

In this regard, the authors now state that “Hif1α KO embryos show defects in somites and the neural tube/fold, which are in agreement with our results of the beneficial effects of hypoxia on enhancing the somite and notochord signatures (Fig. 7). However, the Hif1α KO mouse embryos also show severe vascularization and placental defects, complicating the interpretation of their phenotype (Kozak et al., 1997).”
This statement needs to be refined, I think. As far as I know, there is no vascularization phenotype described at day 8.5dpc in Hif1a KO embryos, this phenotype manifests later. Moreover, from the in vivo KO data, there is no evidence that germ layer specification or gastrulation is affected. To conclude that the in vitro expression analysis (Fig.7) is agreeing with the KO in vivo phenotype appears therefore misleading. Again, while this can certainly be addressed by rephrasing this paragraph, it re-enforces the importance to have clear functional evidence for the role of Hif1 for the observed phenotype, in vitro. Once this is clarified, only then the comparison to the in vivo Hif1a KO phenotype is actually relevant.

We removed the argument about vascularization now. As the Reviewer points out, and as we mention in the text, germ layer formation and specification is not affected. Here we would like to mention again that we identify hypoxia as an environmental regulator and enhancer of tissue diversification. We do not claim, nor is it true, that hypoxia or HIF1a are required for germ layer or body axis formation or gastrulation. The fact that we do not observe direct HIF1a binding at primitive streak genes would point to this anyway. We show, however, that the complete hypoxia response and HIF1a chemical induction (which likely induces other downstream targets) induces primitive streak genes. All in all, we remain of the opinion that hypoxia is a major environmental factor shaping - but not required for – cell type diversification.
Secondly, the classical HIF1a KO embryo studies from the last decades could not thoroughly investigate the transcriptional profiles of each cell type. Thus, although the phenotype of the embryo at E8.5 looks normal, we cannot know whether this is via convergence or compensation at late gastrulation. Whether HIF1a KO embryos manifest any transcriptional perturbations needs to be seen at the single cell level, which is not the focus of our study.

3)The authors have not addressed my concern about their conclusions on the role of glycolysis. “The metabolic shift to glycolysis does not contribute to the hypoxia-mediated early primitive streak signature” is in my view a conclusion that is not supported sufficiently by their data. As I

© 2022. Published by The Company of Biologists under the terms of the Creative Commons Attribution License (https://creativecommons.org/licenses/by/4.0/).

outlined previously, the issue I see is that the authors do not perturb glycolysis directly. They only apply a perturbation strategy using the mitochondrial inhibitor FCCP (and combine this treatment with hypoxia treatment, complicating the expected impact on glycolysis further). How and if this affects glycolysis is unclear and not investigated, beyond measurement of lactate (which can, however, be altered for several reasons). Along this line, it is of note that upon FCCP treatment, Pfkfb3 expression is not increased (see Figure 2 G), even though it is known that increased flux will lead to increased Pfkfb3 expression. Without doubt, more direct manipulation of glycolytic flux is needed and feasible in this simplified in vitro setting.

Based on the Reviewer's suggestion, we now revised the claim about the lack of contribution of glycolysis to the phenotype. We now only relate the conclusions to altered lactate production, and its inability to replicate the observed effects. Revised text reads as:

'Altering lactate production does not replicate the hypoxia-mediated early primitive streak signature
A major component of the hypoxia response is the shift of cellular metabolism from oxidative phosphorylation to glycolysis. Indeed, metabolic rewiring constitutes the main hypoxia response in ESCs at the protein level (Fig. 2E). Metabolic pathways not only determine ways of energy utilization but also impact cellular states and developmental phenomena (Bulusu et al., 2017; Hu et al., 2020; Oginuma et al., 2017; Rodriguez-Terrones et al., 2020). Therefore, we next set out to dissect the influence of lactate production from other hypoxia-mediated events in the induction of early primitive streak genes. For this, we treated cells with an inhibitor of oxidative phosphorylation (iOxPhos) (Fig. 2F-H), which led to increased lactate production (Fig. 2F). The expression levels of glycolytic enzymes increase in hypoxia but not upon iOxPhos treatment (Fig. 2F,G). Although increasing lactate production, iOxPhos treatment in normoxia or in hypoxia does not by itself induce Wnt3, T, or Eomes expression, nor does it affect other developmental or pluripotency-associated genes (Fig. 2H). Overall, these results suggest that the induction of developmental genes in hypoxia cannot be replicated only by altering the glycolytic output.'

Third decision letter

MS ID#: DEVELOP/2022/200679

MS TITLE: Hypoxia induces an early primitive streak signature, enhancing spontaneous elongation and lineage representation in gastruloids

AUTHORS: Natalia Lopez-Anguita, Seher Ipek Gassaloglu, Maximilian Stoetzel, Adriano Bolondi, Deniz Conkar, Marina Typou, Rene Buschow, Jesse Veenvliet, and Aydan Bulut-Karslioglu
ARTICLE TYPE: Research Article

I am happy to tell you that your manuscript has been accepted for publication in Development, pending our standard ethics checks.

© 2022. Published by The Company of Biologists under the terms of the Creative Commons Attribution License (https://creativecommons.org/licenses/by/4.0/).
